# The new definition of obesity: an analysis of a population-based survey in an Andean country

**DOI:** 10.1016/j.lana.2025.101217

**Published:** 2025-08-29

**Authors:** Jamee Guerra Valencia, Akram Hernández-Vásquez, Percy Mayta-Tristán, Lorena Saavedra-Garcia, Rodrigo Vargas-Fernández

**Affiliations:** a Facultad de Ciencias de la Salud, Universidad Privada del Norte, Lima, Peru; b Centro de Excelencia en Investigaciones Económicas y Sociales en Salud, Vicerrectorado de Investigación, Universidad San Ignacio de Loyola, Lima, Peru; c Universidad Científica del Sur, Lima, Peru; d Grupo de Investigación en Nutrición Funcional, Carrera de Nutrición y Dietética, Facultad de Ciencias de la Salud, Universidad San Ignacio de Loyola, Lima, Peru; e Epidemiology and Health Economics Research (EHER), Universidad Científica del Sur, Lima, Peru

**Keywords:** Peru, Obesity, Ethnicity, Body mass index, Waist circumference

## Abstract

**Background:**

Traditional obesity classification based on body mass index (BMI) fails to capture body fat distribution or clinical dysfunction, and may therefore fail to identify people at highest cardiometabolic risk. The Lancet Diabetes & Endocrinology Commission recently proposed a new framework distinguishing preclinical from clinical obesity based on excessive adiposity and clinical dysfunction. We aimed to estimate the prevalence of clinical and preclinical obesity in Peruvian adults using the Commission’s criteria and adapted regional cutoffs, and to generate ethnicity-specific reference curves for waist circumference (WC) and waist-to-height ratio (WHtR).

**Methods:**

This cross-sectional analysis used nationally representative data from 2021 to 2023 Peruvian Demographic and Health Surveys (ENDES), including 84,622 adults aged ≥20 years. Clinical obesity was defined as excess body fat (BMI, WC, or WHtR) plus diabetes or hypertension diagnosis. Preclinical obesity was defined as excess body fat without clinical dysfunction. Age-adjusted prevalence estimates were calculated using four anthropometric criteria and stratified by sex. Ethnicity-specific WC and WHtR reference curves were generated using GAMLSS models, stratified by age and sex.

**Findings:**

A total of 84,622 participants were included in the study. Among these participants, mean age was 44.1 (range: 20–97) years, and 48,300 participants (51.7%) were female. Clinical obesity age-adjusted prevalence ranged from 15.7% to 22.1%, and preclinical obesity from 28.7% to 53.8%, depending on cutoffs used. Up to 13.5% of individuals with normal BMI and 21% of those overweight met criteria for clinical obesity. Women showed the highest prevalence estimates of preclinical obesity, ranging from 33.4% to 65.8%, whereas men reached their highest prevalence (41.3%) when the International Diabetes Federation (IDF) cutoffs were applied. In the case of clinical obesity, women had higher prevalence estimates of clinical obesity when applying the Lancet Commission approach (18.7%) and the Peruvian national guidelines (21.4%). Men showed higher prevalence estimates when using the cutoffs proposed by the Latin American Consortium of Studies in Obesity (LASO) (16.8%) and the IDF (22.8%). Reference curves showed that Quechua-Aymara individuals had lower WC and WHtR values compared to Afro-Peruvian and other groups at the 97th percentile, in both men and women.

**Interpretation:**

Reliance on BMI alone underestimates a large proportion of clinically relevant cases. Incorporating WC-measurements and clinical dysfunction into diagnostic frameworks could improve identification, prevention, and policy responses to obesity in Peru and similar settings.

**Funding:**

The authors received no financial support.

**Research in context**

**Evidence before this study**

We searched PubMed for studies published in English, Spanish or Portuguese from database inception to April 28, 2025. The search term “clinical obesity” was used to specifically target studies aligned with the new classification proposed by The Lancet Diabetes & Endocrinology Commission. We identified two recent studies that applied the new classification of obesity. One of the studies used the new classification to define the prevalence of clinical obesity in a population-based study in Dubai, while the other aimed to determine the association between remnant cholesterol and obesity phenotypes (preclinical and clinical obesity) using data from the National Health and Nutrition Examination Survey (NHANES). In the Dubai study, the authors also estimated the shift in the body mass index (BMI) required for individuals to be reclassified as having clinical obesity under the new definition. Based on this evidence, no studies to date have applied the new obesity classification in Andean countries.

**Added value of this study**

Our population-based study applied the new classification proposed by The Lancet Diabetes & Endocrinology Commission. We estimated the prevalence of both clinical and preclinical obesity in Peruvian adults using the criteria of the Commission alongside regionally adapted definitions and compared the resulting classification shifts with those based on the previous BMI thresholds. We conducted this analysis using a nationally representative, population-based dataset, enhancing the relevance of our findings for public health practice. We also developed ethnicity-specific reference curves for waist circumference and waist-to-height ratio measures, as current national thresholds may not adequately capture ethnic and altitude-related variability in body composition. This is a relevant aspect in an Andean country such as Peru, where marked geographic and ethnic diversity influences anthropometric patterns and obesity-related risk profiles.

**Implications of all the available evidence**

Our findings highlight the importance of moving beyond BMI-only approaches by incorporating waist circumference and clinical dysfunction to improve obesity classification. Sole reliance on the BMI may result in misclassification, leading to inefficient resource allocation, either by directing interventions toward individuals who may not benefit or by overlooking those who are truly at risk. Moreover, the development of ethnicity-specific reference curves represents an important first step that can inform future validation studies to establish context-appropriate thresholds, and improve the clinical and epidemiological assessment of obesity.

## Introduction

Over recent decades, the debate over whether obesity should be recognised as a disease or only a risk factor for other conditions has remained unresolved.^1^ In clinical settings and epidemiological studies, obesity is typically defined as excessive body fat accumulation, often measured by a body mass index (BMI) equal or greater than 30 kg/m^2.^^2^ However, BMI is not a direct measure of body fat, nor does it account for body fat distribution, potentially leading to misclassification of excess adiposity and result in an over- or underdiagnosis of obesity.^2^

Recently, The Lancet Diabetes & Endocrinology Commission proposed a new classification that distinguishes between preclinical and clinical obesity, based on evidence of organ or tissue impairment and impaired daily functioning.^3^ To mitigate misclassification, the Commission recommends confirming excess adiposity through either direct body composition assessments or a combination of BMI and central adiposity indicators—such as waist circumference (WC), waist-to-hip ratio, or waist-to-height ratio (WHtR). Clinical obesity, in particular, requires not only confirmation of excess body fat but also clinical evaluation to identify related health consequences, similar to the diagnostic approach used for chronic diseases.^3^ Since BMI is widely used in population-based studies to define obesity,^2^ this classification—which considers BMI a surrogate measure—could shift the current obesity estimates and approach, potentially reclassifying 890 million people living with this condition and reshaping healthcare needs and resource allocation.^4^

Central adiposity measures like WC, WHtR, and waist-to-hip ratio are essential for this framework. However, the cutoff values used to define elevated central fat are primarily derived from Caucasian populations.^3^ The elephant is (still) in the room: these thresholds may not be appropriate for diverse populations, as body composition and fat distribution vary considerably across age, sex, ethnicity, and regions, raising concerns about their applicability in diverse demographic contexts.^5^ In Latin American and Caribbean (LAC), the rise in obesity rates is driven by a complex interplay of structural, contextual, and individual-level factors.^6^ Furthermore, LAC is one of the most ethnically diverse regions globally, yet specific anthropometric cutoffs in its ethnic groups remain unexplored.^6^

In Peru, a country with diverse geography and a multiethnic population, the prevalence of obesity has increased by 5.6 percentage points between 2014 and 2023,^7^ making it a key focus of national research agendas.^8^ However, prevalence estimates differ depending on the anthropometric indicators applied,^9^ and current criteria remain insufficiently adapted to capture ethnic variability. Ethnic differences, in particular, introduce further variability in body fat distribution^10^ potentially compromising the accuracy of obesity classification when applying universal thresholds. We therefore aimed to estimate the prevalence of clinical and preclinical obesity in the adult Peruvian population using the new classification proposed by The Lancet Diabetes & Endocrinology Commission. Furthermore, as recognized by the Commission, age, sex and ethnicity or country specific thresholds are needed to reduce misclassification,^3^ for which we also aimed to examine obesity estimates variation when using adapted classification criteria that reflect regional or national anthropometric characteristics to better characterize obesity in a diverse population such as the Peruvian. Moreover, as noted, WC and WHtR vary by age, sex, and ethnicity,^5^ particularly in multiethnic populations like Peru, where differences in fat distribution may influence obesity-related risk and support the need for ethnicity-specific reference curves based on nationally representative data. In the absence of validated regional thresholds, current guidelines from international institutions such as the International Diabetes Federation (IDF) recommend using South Asian cutoffs for South American populations, an approach that may not reflect local anthropometric and metabolic characteristics.^11^ Therefore, we also provide age- and ethnicity-specific reference curves for WC and WHtR to evaluate intra-population variability. These findings highlight the necessity of establishing and validating locally relevant cutoff points to ensure accurate health assessments. The data presented here may serve as a foundational resource for future research aimed at refining obesity risk stratification in multiethnic populations, such as that of Peru.

## Methods

### Study design and setting

This cross-sectional analytical study was based on secondary data from the Peruvian Demographic and Health Survey (ENDES, in Spanish), conducted by the National Institute of Statistics and Informatics of Peru (INEI, in Spanish). The ENDES is a nationally representative household survey that collects demographic, health, and nutritional data across the country. It employs a two-stage stratified probabilistic sampling design, first selecting clusters (primary sampling units) and then households within each cluster. Stratification is done at the department level and by urban-rural areas, ensuring representation at national and subnational levels. The current study compiled the data from the ENDES conducted in 2021, 2022, and 2023. We adhered to the STROBE (Strengthening the Reporting of Observational Studies in Epidemiology) (see Supplementary Material).

Peru is a South American country characterized by diverse geographic landscapes, including coastal regions, Andean highlands, and Amazonian rainforests, which influence population distribution and health disparities. The nation has a multiethnic population, with a predominant Indigenous Amerindian ancestry, particularly in the highlands and Amazon, and a higher proportion of European ancestry along the coast. Additionally, African and Asian genetic contributions reflect historical migrations, shaping Peru’s complex demographic landscape.^12^ Peru is divided into 24 political-administrative regions, which are geographically distributed across three natural areas: the Coast, the Highlands, and the Amazon. The Coast is the most urbanised and industrialised, with better access to healthcare services and higher average income levels. The highlands are predominantly rural, with low population density and higher levels of poverty. The Amazon region is geographically remote, with poverty levels comparable to those in the highlands. Both the highlands and the jungle have limited access to basic infrastructure and healthcare services.^13^

### Participants

The study population included adults aged 20 years or older. The age threshold was based on the World Health Organization (WHO) guidelines, which recommend applying standard BMI cutoffs for the classification of nutritional status (underweight, normal weight, overweight, and obesity) only to adults aged 20 years and above.^2^ Those with incomplete information regarding the anthropometric measurements (weight, height, WC), clinical relevant variables (blood pressure measurement, self-reported diabetes mellitus diagnosis), and ethnicity were excluded. Participants with extreme values (i.e., BMI < 13 kg/m^2^ and BMI > 70 kg/m^2^) were also excluded.

### Variables and measurements

#### Preclinical and clinical obesity

The main outcome in the present analysis was the obesity as clinical or preclinical according to the new definition provided by The Lancet Diabetes & Endocrinology Commission in 2025.^3^ Preclinical obesity was defined as excessive body fat and no ongoing illness. Clinical obesity was defined as the presence of excessive body fat and ongoing organ dysfunction and/or reduced ability to conduct daily activities.^3^ Excessive body fat was determined as: i) high BMI plus at least one high WC-based measurement (i.e., high WC or high WHtR) or ii) WC and high WHtR or iii) extreme BMI. Proposed recommended cutoffs by The Lancet Diabetes & Endocrinology Commission were used for BMI, WC, and WHtR to reflect their original approach for confirmation of excessive body fat. Additionally, other specific BMI, WC, and WHtR cutoffs were used to reflect the Peruvian national context. For the ongoing illness, signs/symptoms of organ dysfunction and individual limitations of day-to-day activities were assessed.

Four reference documents and guidelines were used to establish the cutoff points of anthropometric measures to construct the adapted confirmation of excessive body fat (i.e., obesity) (Table 1). These include recommendations from The Lancet Diabetes & Endocrinology Commission on Clinical Obesity,^3^ technical guidelines from the Peruvian National Institute of Health,^14^^,^^15^ epidemiological criteria from the Latin American Consortium of Studies in Obesity (LASO),^16^ and international standards from the International Diabetes Federation (IDF).^11^ Therefore, four different approaches for the confirmation of the obesity presence (i.e., excessive body fat) were constructed. These approaches are presented in Table 1.

**Table 1:** Obesity confirmation criteria based on The Lancet Diabetes & Endocrinology Commission on Clinical Obesity and adapted regional anthropometric cutoffs.

Obesity confirmation approach	Anthropometric thresholds required for confirmation				
	Criterion 1		Criterion 2		Criterion 3
**Approach 1** Based on The Lancet Diabetes & Endocrinology Commission on Clinical Obesity^a^	BMI ≥ 30 kg/m^2^ (≥20 years old) AND [WC ≥ 88 cm (women) WC ≥ 102 cm (men) OR WHtR ≥ 0.5]	**OR**	WC ≥ 88 cm (women) WC ≥ 102 cm (men) AND WHtR ≥ 0.5	**OR**	BMI ≥ 40 kg/m^2^ (≥20 years old)
**Approach 2** Adapted from Technical Guidelines of the National Institute of Health (Peru)	BMI ≥ 30 kg/m^2^ (20–59 years) BMI ≥ 32 kg/m^2^ (≥60 years) AND [WC ≥ 80 cm (women) WC ≥ 94 cm (men) OR WHtR ≥ 0.5]	**OR**	WC ≥ 80 cm (women) WC ≥ 94 cm (men) AND WHtR ≥ 0.5	**OR**	BMI ≥ 40 kg/m^2^ (≥20 years old)
**Approach 3** Adapted from The Latin American Consortium of Studies in Obesity (LASO^b^)	BMI ≥ 30 kg/m^2^ (20–59 years) BMI ≥ 32 kg/m^2^ (≥60 years) AND [WC ≥ 94 cm (women) WC ≥ 97 cm (men) OR WHtR ≥ 0.5]	**OR**	WC ≥ 94 cm (women) WC ≥ 97 cm (men) AND WHtR ≥ 0.5	**OR**	BMI ≥ 40 kg/m^2^ (≥20 years old)
**Approach 4** Adapted from The International Diabetes Federation (IDF)	BMI ≥ 30 kg/m^2^ (20–59 years) BMI ≥ 32 kg/m^2^ (≥60 years) AND [WC ≥ 80 cm (women) WC ≥ 90 cm (men) OR WHtR ≥ 0.5]	**OR**	WC ≥ 80 cm (women) WC ≥ 90 cm (men) AND WHtR ≥ 0.5	**OR**	BMI ≥ 40 kg/m^2^ (≥20 years old)

BMI: Body mass index. WC: Waist circumference. WHtR: Waist-to-height ratio.

a For Approach 1, BMI ≥ 30 kg/m^2^ was adopted for Criterion 1 based on Appendix 2.5, Table 3 from Rubino et al., 2025 (The Lancet Diabetes & Endocrinology Commission). Although the main text referred to BMI > 30 kg/m^2^, the appendices used ≥30. BMI ≥ 40 kg/m^2^ was used for Criterion 3 due to inconsistencies in the main text regarding the threshold for “severe obesity”.

b Extracted from Table 6 of Herrera et al., 2009. WHtR ≥ 0.5 was applied to Criteria 1 and 2 in accordance with the original cut-off proposed by Ashwell & Hsieh (2005), and to ensure consistency given the discrepancies observed in the supplementary references.

According to the Lancet Diabetes & Endocrinology Commission clinical obesity requires the presence of signs/symptoms of organ dysfunction or individual limitations of day-to-day activities.^3^ In the current study, clinical obesity was considered as present when any of the aforementioned excessive body fat criteria was accomplished plus at least one of the following clinical conditions: blood hypertension or self-reported diabetes mellitus (DM) diagnosis. When excessive body fat was present but without hypertension or DM, preclinical obesity was considered as present.

#### Blood hypertension and diabetes mellitus

Hypertension was defined as the systolic blood pressure (SBP) average ≥ 140 mmHg and/or the diastolic blood pressure (DBP) average ≥ 90 mmHg, or when the individual reported having been previously diagnosed with ‘hypertension’ or ‘high blood pressure’ by a physician (Has a doctor ever told you that you have ‘hypertension’ or ‘high blood pressure’?). In the ENDES survey, two consecutive measurements of SBP and DBP were taken by trained personnel using an automatic blood pressure monitor (Model HEM-7113). The first measurement was taken after 5 min of rest, and the second was taken 2 min later.^17^ The average of both measurements was used to determine each participant's blood pressure.

The self-report of previous diagnosis of DM was defined as the condition in which the participant reported having been diagnosed with diabetes or “high blood sugar” by a physician. This was determined based on an affirmative response to the question: “Has a doctor ever told you that you have diabetes or ‘high blood sugar'?”. Participants responding “yes” to the question were considered as having diabetes.

#### Stratification variables

##### Sex

Preclinical and clinical prevalence estimates were stratified by sex as recorded by interviewer observation in the ENDES survey.

##### Ethnicity

Following previous recommendations, the self-reporting ethnicity approach was chosen as an ethnicity indicator.^18^ Those reporting self identification as Quechua or Aymara were categorized as “Quechua-Aymara”, while those who self-identified as Black, “moreno,” “zambo,” “mulato,” Afro-Peruvian, or of African descent were classified as “Afro-Peruvian”. Individuals who identified as White, Mestizo, Amazonian native or Indigenous, belonging to or part of another Indigenous or original people, or other, were included in the “Other” category”.

### **Statistical analysis**

Descriptive statistics were applied to report the sociodemographic and health characteristics of the participants. The age-adjusted weighted prevalence of preclinical and clinical obesity was estimated using the direct method based on the World Health Organization (WHO) standard population,^19^ and reported based on the four reference documents and guidelines. Using the age-adjusted estimates, both preclinical and clinical obesity were mapped to visualize their geographic distribution. Age-adjusted estimates were also used to construct the Sankey diagram ilustrating the transition from traditional obesity measures (i.e., BMI alone) to the new definition of preclinical and clinical obesity. This transition was also assessed based on the four reference documents and guidelines.

To assess the variations and distribution of WC and WHtR by age and ethnic group, we undertook the GAMLSS (Generalized Additive Models for Location, Scale, and Shape) methodology. This methodological approach enables a more flexible and robust statistical analysis of anthropometric variables. We employed the BCCG (Box-Cox Cole Green), a statistical technique that allows modelling asymmetric and non-normal distributions, which are frequently observed in anthropometric data. The analysis focused on estimating the 3rd, 50th, and 97th percentiles of the anthropometric variables evaluated. Separate models were fitted based on age and sex (ranging from 20 to 97 years) for each ethnic group (Quechua-Aymara, Afro-Peruvian, and Other). These estimates were used to generate ethnicity-specific reference curves across sex and age groups, capturing the intricate variations in body measurements.

Analyses were conducted in R version 4.5.1 and Rstudio version 2025.5.1.513 using various packages for survey data management and statistical analyses. Sampling weights provided by the ENDES were applied to ensure that the estimates were representative of the national population.

### **Ethical considerations**

The present study used de-identified and publicly available data from the ENDES. As the dataset contains no personal identifiers, the research was classified as non-human subjects research, and IRB approval was not required. The databases are freely accessible through the INEI website (https://proyectos.inei.gob.pe/microdatos/).

### Role of funding source

This study did not have any funding.

## Results

We included 84,622 participants in the analysis. Of them, 51.7% were women, and the mean age was 44.1 years (range: 20–97). Among the sociodemographic characteristics, more than a quarter of the participants self-identified as belonging to the Quechua or Aymara ethnic groups, 81.5% lived in urban areas and 41.4% had a high school education (Table 2). More than a quarter of the participants resided at 1500 m or higher above sea level (m.a.s.l.). Additional details on the sample characteristics are provided in Table 2.

**Table 2:** Descriptive characteristics of the adult population in Peru, 2021–2023.

Characteristic	n	% (95% CI)
Sex		
Male	36,322	48.3 (47.7–48.8)
Female	48,300	51.7 (51.2–52.3)
Age group		
Young adult (18–29)	21,760	23.1 (22.7–23.5)
Adult (30–59)	50,147	57.2 (56.7–57.8)
Older (60 or older)	12,715	19.7 (19.2–20.1)
Ethnicity		
Quechua or Aymara	26,662	26.3 (25.8–26.8)
Afro-Peruvian	8921	11.6 (11.2–11.9)
Other	45,858	62.1 (61.6–62.7)
Wealth quintile		
Poorest	27,581	18.5 (18.2–18.9)
Poor	21,305	19.8 (19.4–20.3)
Middle	15,532	20.8 (20.3–21.3)
Rich	11,813	20.6 (20.1–21.1)
Richest	8391	20.2 (19.6–20.8)
Education		
Up to primary	23,287	23.6 (23.2–24.1)
Secondary	35,504	41.4 (40.9–42.0)
Higher	25,831	34.9 (34.4–35.5)
Natural region		
Coast	33,503	63.1 (62.6–63.7)
Highlands	30,695	24.2 (23.7–24.8)
Jungle	20,424	12.6 (12.2–13.0)
Area of residence		
Urban	54,388	81.5 (81.2–81.8)
Rural	30,234	18.5 (18.2–18.8)
Altitude of residence		
0–499 m.a.s.l.	41,402	66.4 (65.7–67.1)
500–1499 m.a.s.l.	9937	7.6 (7.0–8.1)
1500–2999 m.a.s.l.	13,452	11.3 (10.9–11.8)
3000 m.a.s.l. or more	19,831	14.7 (14.2–15.2)

Estimates include the weights and ENDES sample specifications.

m.a.s.l.: meters above sea level. CI: confidence interval.

Using the Commission anthropometric cutoffs for diagnosing obesity, the age-adjusted prevalence estimates revealed that approximately one-third of the sample would be classified as preclinical obese, while 16.0% would be categorized as clinically obese. When adapting the Commission approach with the anthropometric cutoffs of the Peruvian national guidelines (i.e., BMI age-specific cutoff and WC sex-specific cutoff), both preclinical and clinical obesity prevalence rose to 48.8%, and 20.6%, respectively. Using WC sex-specific cutoffs from LASO and IDF, the prevalence of preclinical and clinical obesity was 28.7% and 15.7% for LASO, and 53.8% and 22.1% for IDF, respectively (Table 3). Sex stratified age-adjusted prevalence estimates showed that women had a high prevalence of preclinical obesity which ranged from 33.4% when using the LASO cutoffs to 65.8% when applying both the Peruvian national guidelines and the IDF cutoffs. In contrast, men had the highest prevalence of preclinical obesity when classified using the IDF cutoffs (41.3%). Overall, women showed a higher prevalence of clinical obesity compared to men when using the anthropometric cutoffs proposed by the Lancet Commission and the Peruvian national guidelines, but a lower prevalence when classified according to the LASO and IDF criteria (Table 3).

**Table 3:** Age-adjusted prevalence of preclinical and clinical obesity using different obesity confirmation approach.

Classification	n	Overall age-adjusted prevalence% (95% CI)	Male age-adjusted prevalence% (95% CI)	Female age-adjusted prevalence% (95% CI)
The Lancet Diabetes & Endocrinology Commission On Clinical Obesity^a^				
No obesity	45,043	51.3 (50.7–51.8)	71.1 (70.3–71.9)	32.9 (32.2–33.5)
Preclinical obesity	29,540	32.8 (32.3–33.2)	16.1 (15.4–16.7)	48.4 (47.7–49.1)
Clinical obesity	10,039	16.0 (15.6–16.4)	12.9 (12.3–13.5)	18.7 (18.1–19.3)
Adapted to the technical guidelines of the National Institute of Health^b^				
No obesity	27,214	30.6 (30.1–31.1)	49.4 (48.6–50.2)	12.8 (12.4–13.3)
Preclinical obesity	44,282	48.8 (48.3–49.3)	30.9 (30.1–31.6)	65.8 (65.1–66.4)
Clinical obesity	13,126	20.6 (20.2–21.1)	19.7 (19.0–20.4)	21.4 (20.8–22.0)
Adapted to the Latin American Consortium of Studies in Obesity (LASO)^c^				
No obesity	50,615	55.6 (55.1–56.1)	59.6 (58.8–60.3)	51.9 (51.2–52.6)
Preclinical obesity	24,311	28.7 (28.2–29.1)	23.6 (22.9–24.3)	33.4 (32.7–34.1)
Clinical obesity	9696	15.7 (15.3–16.2)	16.8 (16.2–17.5)	14.7 (14.2–15.2)
Adapted to the International Diabetes Federation (IDF)^d^				
No obesity	22,065	24.1 (23.6–24.5)	35.9 (35.2–36.6)	12.8 (12.4–13.3)
Preclinical obesity	48,383	53.8 (53.3–54.3)	41.3 (40.5–42.1)	65.8 (65.1–66.4)
Clinical obesity	14,174	22.1 (21.7–22.6)	22.8 (22.1–23.5)	21.4 (20.8–22.0)

Estimates include the weights and ENDES sample specifications.

Estimates are age-adjusted for all adults aged 20 and over by the direct method to the population of the World Health Organization.

For each of the classification presented above, the following criteria for defining obesity was used.

a Any of the following three criteria: i) BMI ≥ 30 kg/m^2^ for individuals aged ≥ 20 years and either a waist circumference ≥ 88 cm for women or ≥102 cm for men, or a waist-to-height ratio ≥ 0.5; or ii) waist circumference ≥ 88 cm for women or ≥102 cm for men and a waist-to-height ratio ≥ 0.5; or iii) BMI ≥ 40 kg/m^2^ for individuals aged ≥ 20 years.

b Any of the following three criteria: i) BMI ≥ 30 kg/m^2^ for individuals aged 20–59 years or BMI ≥ 32 kg/m^2^ for adults aged ≥ 60 years, and either a waist circumference ≥ 80 cm for women or ≥94 cm for men, or a waist-to-height ratio ≥ 0.5; or ii) waist circumference ≥ 80 cm for women or ≥94 cm for men and a waist-to-height ratio ≥ 0.5; or iii) BMI ≥ 40 kg/m^2^ for individuals aged ≥ 20 years.

c Any of the following three criteria: i) BMI ≥ 30 kg/m^2^ for individuals aged 20–59 years or BMI ≥ 32 kg/m^2^ for adults aged ≥ 60 years, and either a waist circumference ≥ 94 cm for women or ≥97 cm for men, or a waist-to-height ratio ≥ 0.5; or ii) waist circumference ≥ 94 cm for women or ≥97 cm for men and a waist-to-height ratio ≥ 0.5; or a iii) BMI ≥ 40 kg/m^2^ for individuals aged ≥ 20 years.

d Any of the following three criteria: i) BMI ≥ 30 kg/m^2^ for individuals aged 20–59 years or BMI ≥ 32 kg/m^2^ for adults aged ≥ 60 years, and either a waist circumference ≥ 80 cm for women or ≥90 cm for men, or a waist-to-height ratio ≥ 0.5; or ii) waist circumference ≥ 80 cm for women or ≥90 cm for men and a waist-to-height ratio ≥ 0.5; or iii) BMI ≥ 40 kg/m^2^ for individuals aged ≥ 20 years. Clinical obesity was defined as the presence of obesity and at least one of the following conditions: hypertension or self-reported diagnosis of diabetes mellitus.

Additional detail of preclinical and clinical obesity prevalence estimates using the Commission anthropometric criteria and by sociodemographic and geographic characteristics is shown in Table 4. When disaggregating the age-adjusted prevalence by sociodemographic and geographic characteristics, both preclinical and clinical obesity were more prevalent among individuals with a higher wealth index, urban residents, and people living in the coastal region, while prevalence was lower among those living at 3000 m.a.s.l. or more. Regarding sex stratification, lower prevalence was observed in men than women for both preclinical for all the sample characteristics.

**Table 4:** Age-adjusted prevalence of preclinical and clinical obesity using the Lancet Diabetes & Endocrinology Commission on Clinical Obesity anthropometric cutoffs by sample characteristics.

Characteristic	Overall age-adjusted prevalence			Male age-adjusted prevalence			Female age-adjusted prevalence		
	No obesity% (95% CI)	Preclinical obesity% (95% CI)	Clinical obesity% (95% CI)	No obesity% (95% CI)	Preclinical obesity% (95% CI)	Clinical obesity% (95% CI)	No obesity% (95% CI)	Preclinical obesity% (95% CI)	Clinical obesity% (95% CI)
Age group									
Young adult (18–29)	68.4 (67.4–69.4)	28.1 (27.2–29.0)	3.5 (3.1–3.9)	82.9 (81.6–84.1)	12.9 (11.8–14.0)	4.3 (3.6–5.0)	54.1 (52.8–55.4)	43.2 (41.9–44.5)	2.7 (2.3–3.2)
Adult (30–59)	45.4 (44.7–46.1)	38.9 (38.2–39.5)	15.7 (15.1–16.3)	67.0 (66.0–68.1)	19.2 (18.3–20.0)	13.8 (13.0–14.6)	25.4 (24.7–26.2)	57.1 (56.2–58.1)	17.4 (16.7–18.2)
Older (60 or older)	46.4 (45.0–47.8)	20.0 (18.9–21.0)	33.6 (32.3–34.9)	67.9 (65.8–69.9)	10.6 (9.3–11.9)	21.5 (19.8–23.3)	27.4 (25.8–28.9)	28.4 (26.8–30.0)	44.3 (42.4–46.1)
Ethnicity									
Quechua or Aymara	53.4 (52.5–54.3)	33.1 (32.2–34.0)	13.5 (12.8–14.2)	74.6 (73.3–75.9)	14.2 (13.2–15.3)	11.1 (10.1–12.2)	33.9 (32.8–35.0)	50.6 (49.3–51.8)	15.5 (14.6–16.5)
Afro-Peruvian	50.3 (48.9–51.7)	32.8 (31.5–34.1)	16.9 (15.7–18.0)	71.9 (70.0–73.9)	16.3 (14.7–17.9)	11.8 (10.3–13.3)	29.9 (28.2–31.6)	49.3 (47.4–51.2)	20.8 (19.2–22.4)
Other	50.5 (49.8–51.2)	32.6 (31.9–33.2)	16.9 (16.4–17.5)	69.5 (68.5–70.5)	16.7 (15.9–17.5)	13.8 (13.0–14.6)	32.9 (32.0–33.7)	47.4 (46.5–48.3)	19.8 (19.0–20.5)
Wealth quintile									
Poorest	65.2 (64.4–66.0)	26.5 (25.8–27.2)	8.3 (7.8–8.7)	88.4 (87.6–89.2)	7.5 (6.9–8.2)	4.1 (3.6–4.6)	42.6 (41.5–43.6)	45.0 (44.0–46.1)	12.4 (11.6–13.1)
Poor	51.1 (50.1–52.2)	33.9 (33.0–34.9)	14.9 (14.1–15.7)	74.2 (72.8–75.5)	15.2 (14.1–16.3)	10.6 (9.6–11.6)	29.5 (28.4–30.7)	51.5 (50.1–52.8)	19.0 (17.8–20.2)
Middle	47.1 (45.9–48.3)	34.6 (33.5–35.7)	18.3 (17.3–19.2)	67.4 (65.6–69.1)	17.6 (16.2–18.9)	15.1 (13.7–16.5)	28.8 (27.5–30.1)	50.2 (48.7–51.8)	21.0 (19.7–22.2)
Rich	46.5 (45.3–47.8)	34.5 (33.3–35.7)	18.9 (17.9–20.0)	64.9 (63.0–66.7)	18.8 (17.3–20.3)	16.3 (14.8–17.8)	30.3 (28.8–31.7)	48.6 (46.8–50.3)	21.1 (19.8–22.5)
Richest	48.1 (46.6–49.5)	33.2 (31.9–34.5)	18.7 (17.7–19.8)	61.5 (59.4–63.6)	20.6 (18.9–22.3)	17.8 (16.2–19.5)	35.6 (33.8–37.3)	45.1 (43.2–46.9)	19.4 (17.9–20.8)
Education									
Up to primary	50.8 (49.7–51.9)	35.6 (34.5–36.7)	13.6 (13.0–14.3)	81.3 (79.7–82.9)	11.1 (9.6–12.6)	7.6 (6.7–8.5)	31.9 (30.7–33.2)	50.8 (49.5–52.2)	17.2 (16.4–18.0)
Secondary	50.5 (49.6–51.4)	32.4 (31.6–33.2)	17.1 (16.3–17.9)	70.7 (69.5–71.9)	15.9 (15.1–16.8)	13.4 (12.5–14.3)	28.1 (27.1–29.1)	50.2 (48.9–51.4)	21.7 (20.6–22.9)
Higher	50.9 (49.8–52.0)	32.1 (31.2–33.0)	17.0 (16.1–17.9)	66.3 (64.8–67.7)	18.1 (17.0–19.1)	15.7 (14.4–16.9)	34.7 (33.3–36.0)	46.6 (45.1–48.0)	18.8 (17.5–20.0)
Natural region									
Coast	47.1 (46.3–47.9)	34.4 (33.7–35.1)	18.5 (17.9–19.1)	65.6 (64.4–66.7)	18.7 (17.8–19.6)	15.7 (14.9–16.6)	30.0 (29.1–30.9)	49.1 (48.1–50.2)	20.9 (20.1–21.7)
Highlands	58.8 (58.0–59.5)	30.0 (29.3–30.7)	11.2 (10.7–11.7)	81.6 (80.6–82.6)	10.2 (9.5–11.0)	8.2 (7.5–8.9)	38.4 (37.5–39.3)	47.7 (46.8–48.7)	13.8 (13.2–14.5)
Jungle	57.7 (56.8–58.5)	29.8 (29.0–30.5)	12.5 (11.9–13.2)	78.6 (77.6–79.7)	13.6 (12.8–14.5)	7.7 (7.0–8.5)	36.1 (35.0–37.2)	46.2 (45.1–47.4)	17.7 (16.7–18.6)
Area of residence									
Urban	48.2 (47.6–48.8)	34.1 (33.5–34.7)	17.7 (17.2–18.2)	67.3 (66.4–68.2)	17.8 (17.1–18.5)	14.9 (14.2–15.6)	30.7 (30.0–31.4)	49.2 (48.3–50.0)	20.1 (19.5–20.8)
Rural	64.2 (63.5–65.0)	26.8 (26.2–27.5)	8.9 (8.5–9.4)	86.8 (86.0–87.7)	8.3 (7.6–8.9)	4.9 (4.3–5.4)	42.0 (41.0–43.0)	45.1 (44.0–46.1)	12.9 (12.2–13.7)
Altitude of residence									
0–499 m.a.s.l.	48.0 (47.3–48.7)	33.9 (33.2–34.6)	18.1 (17.5–18.7)	66.6 (65.6–67.7)	18.3 (17.5–19.1)	15.1 (14.2–15.9)	30.6 (29.7–31.5)	48.6 (47.6–49.6)	20.8 (20.0–21.6)
500–1499 m.a.s.l.	53.5 (52.2–54.9)	32.8 (31.6–34.0)	13.7 (12.6–14.7)	74.6 (72.7–76.6)	15.6 (14.1–17.1)	9.8 (8.4–11.2)	32.9 (31.2–34.5)	49.7 (48.0–51.4)	17.5 (16.0–18.9)
1500–2999 m.a.s.l.	56.5 (55.4–57.7)	30.3 (29.3–31.3)	13.2 (12.4–14.0)	79.1 (77.5–80.6)	10.6 (9.5–11.7)	10.3 (9.1–11.5)	36.1 (34.7–37.5)	48.2 (46.8–49.7)	15.6 (14.6–16.7)
3000 m.a.s.l. or more	60.6 (59.7–61.6)	29.4 (28.6–30.2)	10.0 (9.4–10.6)	83.6 (82.4–84.7)	9.8 (8.9–10.7)	6.6 (5.8–7.5)	39.9 (38.8–41.1)	47.2 (46.0–48.4)	12.9 (12.1–13.7)

Estimates include the weights and ENDES sample specifications.

Estimates are age-adjusted for all adults aged 20 and over by the direct method to the population of the World Health Organization.

Preclinical and clinical obesity prevalences by sociodemographic and geographic characteristics according to the Lancet Diabetes & Endocrinology Commission on Clinical Obesity definition.

CI: confidence interval; m.a.s.l.: meters above sea level.

**Fig. 1:**
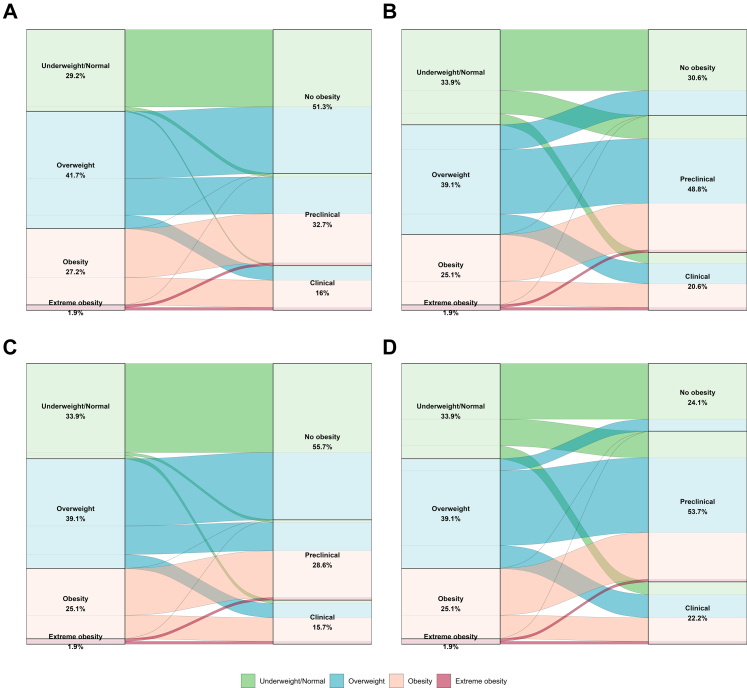
Age-adjusted prevalence and reclassification of obesity status using BMI-based and using different obesity confirmation approach: the Lancet Diabetes & Endocrinology Commission on Clinical Obesity (1A), Peruvian national guidelines (1B), LASO waist circumference cutoffs (1C), and IDF waist circumference cutoffs (1D).

The recategorization of obesity based on BMI cutoffs and the newly Commission anthropometric criteria is illustrated in Fig. 1. In this figure, the left column represents body size classifications based solely on BMI cutoffs, while the right column displays obesity categories according to the new criteria.

In Fig. 1A, The Lancet Diabetes & Endocrinology Commission obesity confirmation approach is applied, showing that most individuals with normal or lower BMI remain non-obese. However, approximately 4% and 1.7% of this group are reclassified as preclinical and clinical obese, respectively. Conversely, no individuals categorized as obese or extremely obese based on BMI are reclassified as non-obese under the Commission criteria.

Fig. 1B–D apply the Peruvian national guidelines' BMI age-specific cutoffs in the left column, while the right column uses different WC sex-specific cutoffs for the newly proposed obesity definition. Using the national guideline cutoffs for BMI and WC (Fig. 1B), approximately 24.5% of individuals in the normal or lower BMI category are classified as preclinical obese, while around 11.5% meet the criteria for clinical obesity. The proportion of normal or lower BMI individuals classified as preclinical or clinical obese varied substantially depending on the WC cutoff applied. When applying the LASO cutoffs (Figure 1C), 2.6% and 3.8% of individuals were classified as preclinical and clinical obese, respectively. In contrast, the IDF cutoffs (Fig. 1D) yielded a higher prevalence, with 28% classified as preclinical obese and 13.5% as clinically obese. Consistent with Fig. 1A, in Fig. 1B–D, no individuals categorized as obese or extremely obese based on BMI were reclassified as non-obese, regardless of the WC cutoffs applied. Importantly, across all anthropometric cutoff, a substantial subset of individuals classified as overweight by BMI, ranging from 11.5% (Fig. 1A) to 21% (Fig. 1D), met the criteria for clinical obesity. Furthermore, when using national guideline (Fig. 1B) and IDF cutoffs (Fig. 1D) for WC, the proportion of overweight individuals reclassified as non-obese were around 23% and 11%, respectively. In contrast, the proportions of overweight individuals classified as non-obese increased to 57% and 61% when using the Commission and LASO cutoffs, respectively. Sex-stratified reclassification are presented in the Supplementary material (see Supplementary material Figs. S1 and S2).

**Fig. 2:**
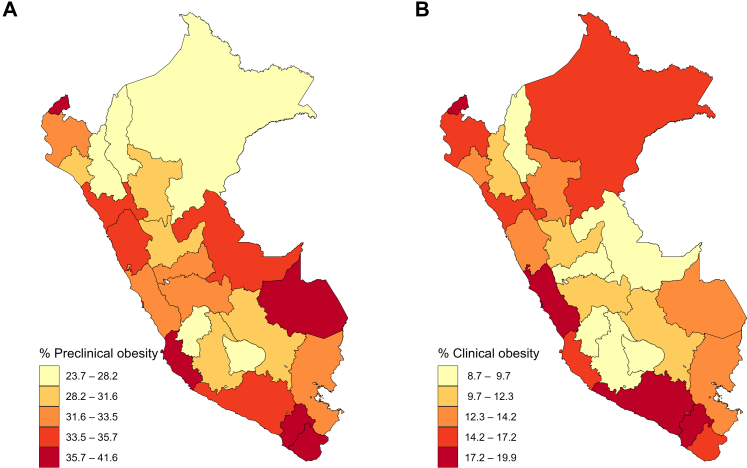
Geographic distribution of age-adjusted preclinical and clinical obesity in Peru using the Lancet Diabetes & Endocrinology Commission on Clinical Obesity definition. (A) Preclinical obestiy. (B) Clinical obesity.

When examining the age-adjusted prevalence of preclinical and clinical obesity with the Commission anthropometric cutoffs at the national political-administrative level, notable regional differences were observed (Fig. 2). For preclinical obesity (Fig. 2A), the highest prevalence was recorded in Tumbes, Ica, Moquegua, and Tacna, all located in the coastal region, and Madre de Dios along with San Martin, in the jungle region. For clinical obesity (Fig. 2B), the highest prevalence was observed in Tumbes, Lima, Callao, Arequipa, and Moquegua, all within the coastal region, and in Loreto, in the jungle region. Sex-specific point estimates for each obesity confirmation approach are available in the Supplementary material (see Supplementary Table S1).

**Fig. 3:**
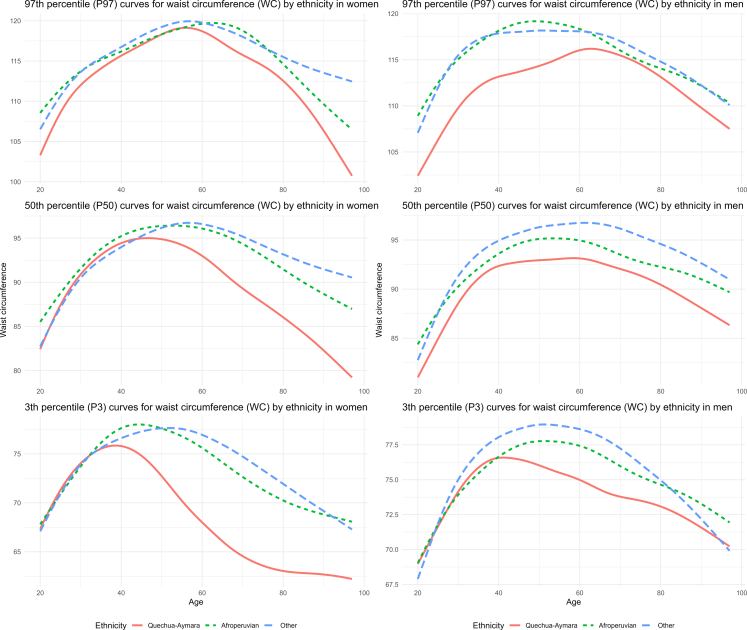
Age-specific percentile curves for waist circumference in Peruvian adults, by ethnicity and sex.

Percentile curves (P3, P50, and P97) for WC showed substantial differences by self-reported ethnicity in both men and women. Across all percentiles, individuals identifying as Quechua-Aymara consistently exhibited lower WC values compared to Afro-Peruvian and other ethnic groups. Age-specific percentiles of WC and WHtR by sex and ethnicity are provided in the Supplementary material (see Supplementary Table S2). These differences were most pronounced at the P97, and the curves revealed a general pattern of increasing WC up to approximately age 50, followed by a gradual decline in both sexes (Fig. 3).

**Fig. 4:**
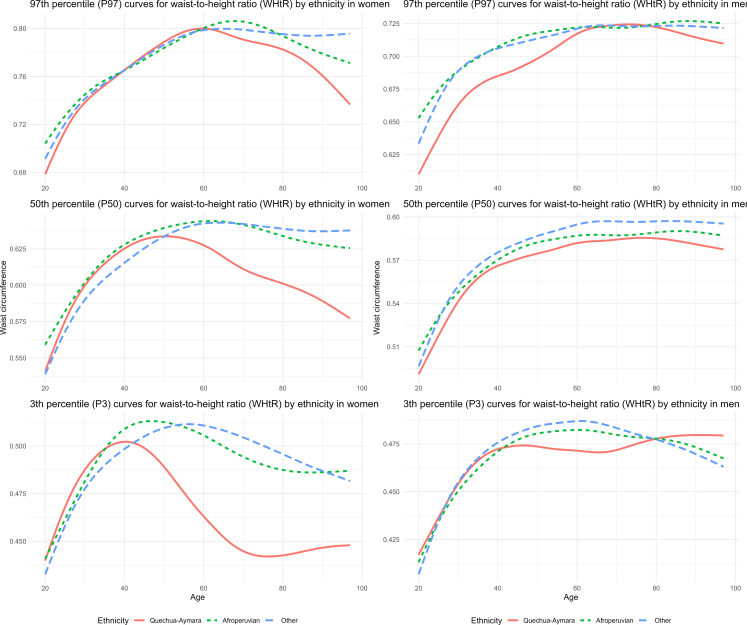
Age-specific percentile curves for waist-to-height ratio (WHtR) in Peruvian adults, by ethnicity and sex.

A similar ethnic pattern was observed for WHtR, with Quechua-Aymara individuals showing lower WHtR values across percentiles, particularly at P97. Among women, the ethnic gap widened notably after age 40, whereas in men the differences remained relatively consistent across age groups (Fig. 4).

## Discussion

This study estimated the prevalence of clinical and preclinical obesity in the Peruvian adults using the newly proposed definition by The Lancet Diabetes & Endocrinology Commission, operationalized through four different anthropometric approaches for the confirmation of excessive body fat. Approximately one-third to over half of adults were classified as preclinical obese, while clinical obesity affected between one-sixth and nearly one-fourth of the population depending on the anthropometric approach used for confirmation. Furthermore, up to 13.5% of individuals with a underweight/normal BMI and 21% of those with overweight met the criteria for clinical obesity. Notably, our findings revealed that prevalence of clinical and preclinical obesity, and the transition from a BMI-based approach to the new definition, varied substantially depending on the anthropometric cutoffs applied.

Historically, BMI has been used in both clinical and epidemiological contexts as the primary anthropometric indicator for assessing obesity^2^ and it remains the standard metric in population-based studies to define the condition.^2^ However, BMI does not distinguish between body fat and lean mass, fails to assess fat distribution, and is not directly linked to clinical dysfunction. In this context, when applying the different anthropometric cutoffs for obesity confirmation as proposed by The Lancet Commission, we found that approximately one-third to more than half of Peruvian adults fall into the preclinical obesity category, and that nearly up to one in four meet the criteria for clinical obesity. These findings highlight the value of adopting a broader framework for obesity classification—one that incorporates not only the amount of body fat but also its clinical consequences.^3^ The identification of clinical and preclinical obesity as distinct entities provides a more precise understanding of disease burden where clinical obesity represents a chronic illness requiring therapeutic intervention, while preclinical obesity represents a heterogeneous phenotype characterised by preserved organ function but increased risk of future complications.^3^ Furthermore, using national demographic projections^20^ our findings suggest that by 2025, over 12.6 million and 5.1 million Peruvian adults aged 20 years or older would meet the criteria for preclinical and clinical obesity, respectively. This underscores the urgent need to rethink both surveillance systems and resource allocation. Recognizing clinical obesity as a diagnosable condition, as proposed by the Lancet Commission,^3^ entails implications for clinical care such as prioritizing access to pharmacological or surgical interventions based on organ dysfunction rather than BMI alone, and supporting timely, evidence-based treatment to prevent irreversible complications.

Age-adjusted prevalence estimates of preclinical and clinical obesity, using the Commission cutoffs, varied notably across political-administrative divisions. Preclinical obesity ranged from 23.7% in Huancavelica to 41.6% in Madre de Dios. In contrast, clinical obesity prevalence ranged from 8.7% in Apurímac to 19.9% in Lima. Notably, the coastal region exhibited consistently high clinical obesity prevalence, ranging from 14.1% to 19.9%, while the jungle region showed greater variability—Ucayali had the lowest prevalence in that region (9%), whereas Loreto reported the highest (15.3%). In the Andean region, most departments showed clinical obesity prevalence below 13.4%, with the notable exception of Arequipa, where 17.5% of the adult population met the criteria for clinical obesity. These geographic patterns may be partially explained by differences in urbanization, altitude, access to healthcare services, among other factors.^6^ Similar disparities across Latin America have been conceptualized within an ecological framework, which highlights how systemic, cultural, and environmental conditions shape obesity risk in the region.^6^ Together, the observed patterns emphasize the need for regionally tailored public health strategies that address local determinants of adiposity including diet, socioeconomic conditions, and health infrastructure. A previous study showed that Peru has one of the lowest levels of healthcare utilization in Latin America, with significant regional inequalities in medical consultations.^21^ It is essential to emphasize that the use of health services among individuals with chronic diseases, such as clinical obesity, requires sustained, long-term, and often complex management approaches. Ensuring equitable access to such services is critical for effective disease management and the prevention of obesity-related complications.

Considering that current policies in Peru for diagnosing, preventing, and treating obesity rely primarily on BMI,^22^ we analysed the classification transitions that would result from applying the Lancet Commission criteria, as well as adapted versions using specific BMI and WC cutoffs. Our results showed that a notable proportion of individuals who would not be classified as obese under BMI alone, particularly those in the underweight/normal or overweight categories meet the criteria for preclinical or even clinical obesity when central adiposity and functional markers are taken into account. However, considerable variability in prevalence estimates was observed depending on the WC cutoffs applied. Among individuals with a underweight/normal BMI, preclinical obesity ranged from 2.6% using LASO criteria to 28% with IDF thresholds. Among those overweight, preclinical obesity ranged from 25.8% to 68% under the same respective cutoffs. Similarly, transitions from underweight/normal BMI and overweight to clinical obesity were also variable, with prevalence reaching up to 13.5% and 21%, respectively, under IDF criteria. Conversely, individuals with a BMI between 30 and 40 kg/m^2^, who are traditionally classified as obese, may not always meet the clinical obesity definition under more stringent definitions such as that proposed by The Lancet Commission. This group may lack overt functional impairment or fall below the WC thresholds used in the different obesity confirmation approaches. As a result, they may not be classified as having clinical obesity, despite having a high BMI. This discordance underscores the clinical heterogeneity within this BMI range and raises relevant considerations regarding risk stratification. Considering national demographic projections^20^ our results suggest that by 2025, between 1.2 and 3 million adults aged 20 years or older, who would be classified as underweight/normal or overweight based on BMI, could remain undiagnosed for clinical obesity if only BMI is used, thus exposing a significant blind spot in current screening and surveillance strategies with economic consequences. In 2018, the annual cost of obesity in Peru (based on BMI classification) was estimated at approximately 1.67 billion soles (about 500 million USD), a figure that exceeded the total public budget allocated that year to the national program for non-communicable diseases.^23^ These findings highlight the urgent need to revise national anthropometric criteria and adopt WC thresholds tailored to local population characteristics. The observed variability in reclassification illustrates the extent to which risk identification can shift depending on the chosen cutoffs, reinforcing the importance of context-specific obesity definitions.

When comparing global age-adjusted prevalence estimates of clinical obesity across the different approaches used in this study, we observed differences of up to 6.4 percentage points. Although any selected WC cutoff led to reclassification in individuals’ BMI-based obesity classification, such discrepancies highlight the policy-level implications of relying on imported cutoffs in contexts with distinct demographic and physiological profiles. Although the use of ethnic-specific cutoffs is internationally recognized,^24^^,^^25^ Peruvian guidelines still rely on WHO reference values.^14^ Despite Peru’s considerable ethnic diversity,^12^^,^^18^ no distinctions have been made in WC cutoffs according to specific ethnic groups, nor have more tailored reference values been adopted for the Latin American population. In contrast, countries such as the United States have adopted ethnicity-specific cutoffs for abdominal adiposity, with different WC thresholds being applied for Asian populations.^26^ While each approach yields different prevalence estimates, none is fully tailored to Peru’s context. National and international cutoffs lack local validation, which may lead to misclassification. This issue extends beyond Peru to other countries with mestizo or multiethnic populations, where imported WC cutoffs may misclassify individuals and undermine prevention efforts. Given the simplicity and feasibility of WC measurement in clinical and public health settings,^27^ it is crucial to establish official cutoffs that align with Peru’s ethnic reality. Implementing evidence-based, population-specific thresholds would enhance the accurate identification of at-risk individuals, allowing for timely interventions and more effective obesity prevention strategies. In line with this, our study generated ethnicity-specific reference curves for WC and WHtR. These curves showed substantial differences across groups where Quechua-Aymara individuals exhibited lower values compared to other groups, particularly at the P97. These findings are consistent with previous evidence from the CANDELA study, which showed that central adiposity measures vary by genomic ancestry in Latin American populations.^10^ In particular, individuals with higher Native American ancestry, such as Peruvian, tended to present greater abdominal fat at lower BMI values, independently of socioeconomic status. This reinforces the need for locally adapted anthropometric criteria that better capture adiposity-related risk across diverse ethnic groups.

Currently, in the absence of validated regional thresholds, institutions such as the IDF recommend applying South Asian WC cutoffs to South American populations.^11^ However, this approach may overlook regional differences in body composition and central adiposity distribution. Our reference curves, which show ethnic variation in WC and WHtR across age, challenge the applicability of such imported thresholds in the Peruvian context and emphasize the need for region-specific standards. Furthermore, the age trajectories of WC observed in our study are broadly consistent with patterns reported in other populations, where WC typically peaks about midlife before declining.^28^^,^^29^ However, some differences arise. For instance the decline observed in Quechua-Aymara men and women is greater than that reported in Venezuelan adults.^29^ Similarly the earlier and more pronounced decline observed in the Quechua-Aymara group suggests potential ethnic or context-specific differences. Regarding WHtR age trajectories, our study identified a non-linear increasing pattern in men, consistent with findings from Colombian adults.^30^ In contrast, among women, we observed a decline beginning around ages 50–60 for the 97th and 50th percentiles, and an earlier decrease at the 3rd percentile. Although Colombian women also showed a non-linear increasing pattern, their sample was limited to individuals under 60 years of age and restricted to the normal BMI range.^30^

From a clinical perspective, these findings have practical implications. For example, in the U.S., WC is routinely measured among individuals with BMI 25–35 kg/m^2^ to refine cardiometabolic risk assessment and guide interventions.^31^ In contrast, Peruvian guidelines recommend skinfolds and bioimpedance,^22^ both methods that are less practical and less widely available due to logistical constraints. Under current policy, nutritional counselling targets those with overweight or obesity defined by BMI, especially in the presence of risk factors.^22^ However, our results suggest that individuals deemed ‘normal weight’ by BMI yet falling within the preclinical or clinical obesity categories based on WC might also warrant early intervention. Since BMI fails to detect excess visceral fat, a key determinant of cardiometabolic risk, incorporating WC into routine screening may improve early identification and management. Rather than endorsing one approach over others, this study highlights how prevalence estimates, and their interpretation, are highly sensitive to the cutoffs applied. This variability along with the absence of outcome-based anthropometric standard thresholds, reinforces the need for locally validated and empirically derived anthropometric cutoffs that can more reliably guide policy, surveillance, and intervention design.

The current study has some limitations that should be acknowledged. The classification of clinical obesity followed the criteria proposed by The Lancet Commission, however we relied on available data for constructing preclinical and clinical obesity, therefore underestimaed prevalence estimates are likely. The excess adiposity was assessed from BMI, WC, and WHtR, but not waist-to-hip ratio as the hip girth is not available in the ENDES. Importantly, this measurement is neither included nor standardized in the Peruvian anthropometric guidelines for adults, which limits its routine collection and may introduce additional error if performed without clear methodological procedures.^14^^,^^15^ For the identification of clinical dysfunction, we relied on self-reported DM and hypertension diagnosis. While this approach does not encompass the full range of organ or tissue impairments outlined in The Lancet Commission, it provides a conservative estimate of clinical obesity prevalence. In this sense, our results likely reflect a lower bound of the true prevalence, as the absence of additional clinical markers in the dataset could lead to underdiagnosis of individuals with other obesity-related complications. Additionally, only two blood pressure measurements were taken in the ENDES. While this may differ from clinical practice guidelines recommending at least three measurements,^32^ the ENDES approach aligns with international standards for population-based hypertension surveillance, which recommend at least two measurements.^33^ The reliance on self-reported diagnoses of DM and hypertension use may introduce recall bias, but these variables have been shown to be reasonably valid in national health surveys.^34^^,^^35^ Importantly, limitations of day-to-day activities was not included in the clinical obesity construction as this information was not available in the dataset. Ethnicity was assessed through self-identification, which may not fully capture genetic ancestry or cultural heterogeneity. Nonetheless, this approach is widely accepted in epidemiological studies^18^ and enables initial exploration of ethnic disparities in adiposity and obesity classification. Finally, it is worth noting that this analysis was based on data from ENDES, a national survey conducted annually with standardized procedures and rigorous sampling methods. ENDES serves as the official source of population-level health indicators in Peru, which reinforces the relevance and generalizability of our findings for national surveillance and policy planning. Furthermore, this also highlights the potential for applying the new clinical and preclinical obesity criteria in large-scale population settings, where their inclusion could enhance the public health surveillance.

Applying the Lancet Commission’s classification revealed that clinical and preclinical obesity are highly prevalent among Peruvian adults, with prevalence estimates varying widely across anthropometric thresholds. A notable share of individuals with normal or overweight BMI met criteria for preclinical or clinical obesity. From a policy perspective, relying solely on BMI leaves many adults with clinically relevant obesity undetected, and prevalence varies widely depending on the WC and WHtR cutoffs applied. Given the marked ethnic differences in central adiposity observed in this study, health authorities should prioritize to develop and validate ethnicity-specific thresholds and incorporate them into revised national obesity criteria, regardless of the conceptual framework adopted. Integrating these tailored cutoffs into national surveillance systems as into clinical practice guidelines may enhance both population-level monitoring and individual-level management. In parallel, obesity monitoring should include functional markers alongside anthropometric indicators to better capture disease burden and guide prioritization of resources.

## Contributors

**JGV** conceived the idea, designed the study, conducted the analysis and wrote the first draft of the manuscript. **AHV** conceived the idea, designed the study, conducted the analysis, produced the tables and figures, and wrote the first draft of the manuscript. **PMT** conceived the idea, and designed the study. **LSG** conceived the idea, and wrote the first draft of the manuscript, edited and provided critical input to improve the manuscript. **RVF** conceived the idea, and wrote the first draft of the manuscript. All authors reviewed the manuscript and contributed important intellectual content to the study and commented critically on the manuscript before its submission. All authors have full access to all the data in the study, accept accountability and approve the submitted version.The corresponding author had the final responsibility for submitting the manuscript for publication.

## Data sharing statement

Data of the ENDES is freely accessible through the National Institute of Statistics and Informatics of Peru (INEI) website (https://proyectos.inei.gob.pe/microdatos/).

## Editor note

The Lancet Group takes a neutral position with respect to territorial claims in published maps and institutional affiliations.

## AI use statement

We acknowledge the use of Large language models (LLMs) to assist with the translation of key terms from English into our first language during the preparation of this manuscript. After using this tool, the authors reviewed and edited the content as needed and took full responsibility for the content of the published article.

## Declaration of interests

No conflict of interest to declare.
