# Supplementary material for: The new definition of obesity: an analysis of a population-based survey in an Andean country
Source: Lancet Reg Health Am. 2025 Aug 29;50:101217. doi: 10.1016/j.lana.2025.101217 (PMC12418883; doi:10.1016/j.lana.2025.101217)
Supplement: Supplementary Figs. S1 and S2 and Tables S1 and S2 [file mmc1.pdf]

Supplementary Material

The new definition of obesity: An analysis of a population-based survey in an Andean country

Table of Contents

Supplementary Material STROBE Statement..……………………………………………………………..2

Supplementary Figure S1………………..…………………………………………………………..4

Supplementary Figure S2………………………..………………………………………………..5

Supplementary Table S1……………………………….………………………………………………6

Supplementary Table S2………………………………………………………….…………...…22

Supplementary Material STROBE Statement—Checklist of items that should be included in reports of cross-sectional studies.

	Item No	Recommendation	Page number
**Title and abstract**	1	(*a*) Indicate the study's design with a commonly used term in the title or the abstract	**1**
		(*b*) Provide in the abstract an informative and balanced summary of what was done and what was found	**3**
**Introduction**			
Background/rationale	2	Explain the scientific background and rationale for the investigation being reported	**4,5**
Objectives	3	State specific objectives, including any prespecified hypotheses	**4,5**
**Methods**			
Study design	4	Present key elements of study design early in the paper	**5**
Setting	5	Describe the setting, locations, and relevant dates, including periods of recruitment, exposure, follow-up, and data collection	**5**
Participants	6	(*a*) Give the eligibility criteria, and the sources and methods of selection of participants	**5**
Variables	7	Clearly define all outcomes, exposures, predictors, potential confounders, and effect modifiers. Give diagnostic criteria, if applicable	**6**
Data sources/ measurement	8*	For each variable of interest, give sources of data and details of methods of assessment (measurement). Describe comparability of assessment methods if there is more than one group	**6,7,8**
Bias	9	Describe any efforts to address potential sources of bias	**6,7,8**
Study size	10	Explain how the study size was arrived at	**5**
Quantitative variables	11	Explain how quantitative variables were handled in the analyses. If applicable, describe which groupings were chosen and why	**6,7,8**
Statistical methods	12	(*a*) Describe all statistical methods, including those used to control for confounding	**8**
		(*b*) Describe any methods used to examine subgroups and interactions	**8**
		(*c*) Explain how missing data were addressed	**NA**
		(*d*) If applicable, describe analytical methods taking account of sampling strategy	**NA**
		(*e*) Describe any sensitivity analyses	**8**
**Results**			
Participants	13*	(a) Report numbers of individuals at each stage of study—eg numbers potentially eligible, examined for eligibility, confirmed eligible, included in the study, completing follow-up, and analysed	**9**
		(b) Give reasons for non-participation at each stage	**5**
		(c) Consider use of a flow diagram	**NA**
Descriptive data	14*	(a) Give characteristics of study participants (eg demographic, clinical, social) and information on exposures and potential confounders	**9**
		(b) Indicate number of participants with missing data for each variable of interest	**NA**
Outcome data	15*	Report numbers of outcome events or summary measures	**9**

Main results	16	(*a*) Give unadjusted estimates and, if applicable, confounder-adjusted estimates and their precision (eg, 95% confidence interval). Make clear which confounders were adjusted for and why they were included	**9,11-17**
		(*b*) Report category boundaries when continuous variables were categorized	**9,11-17**
		(*c*) If relevant, consider translating estimates of relative risk into absolute risk for a meaningful time period	**NA**
Other analyses	17	Report other analyses done—eg analyses of subgroups and interactions, and sensitivity analyses	**19-22**
**Discussion**			
Key results	18	Summarise key results with reference to study objectives	**22**
Limitations	19	Discuss limitations of the study, taking into account sources of potential bias or imprecision. Discuss both direction and magnitude of any potential bias	**25**
Interpretation	20	Give a cautious overall interpretation of results considering objectives, limitations, multiplicity of analyses, results from similar studies, and other relevant evidence	**22-25**
Generalisability	21	Discuss the generalisability (external validity) of the study results	**22-25**
**Other information**			
Funding	22	Give the source of funding and the role of the funders for the present study and, if applicable, for the original study on which the present article is based	**1**

*Give information separately for exposed and unexposed groups.

Note: An Explanation and Elaboration article discusses each checklist item and gives methodological background and published examples of transparent reporting. The STROBE checklist is best used in conjunction with this article (freely available on the Web sites of PLoS Medicine at http://www.plosmedicine.org/, Annals of Internal Medicine at http://www.annals.org/, and Epidemiology at http://www.epidem.com/). Information on the STROBE Initiative is available at www.strobe-statement.org.

Supplementary Figure S1. Age-adjusted prevalence and reclassification of obesity status among men using BMI-based and using different obesity confirmation approach: the Lancet Diabetes & Endocrinology Commission on Clinical Obesity (1A), Peruvian national guidelines (1B), LASO waist circumference cutoffs (1C), and IDF waist circumference cutoffs (1D).

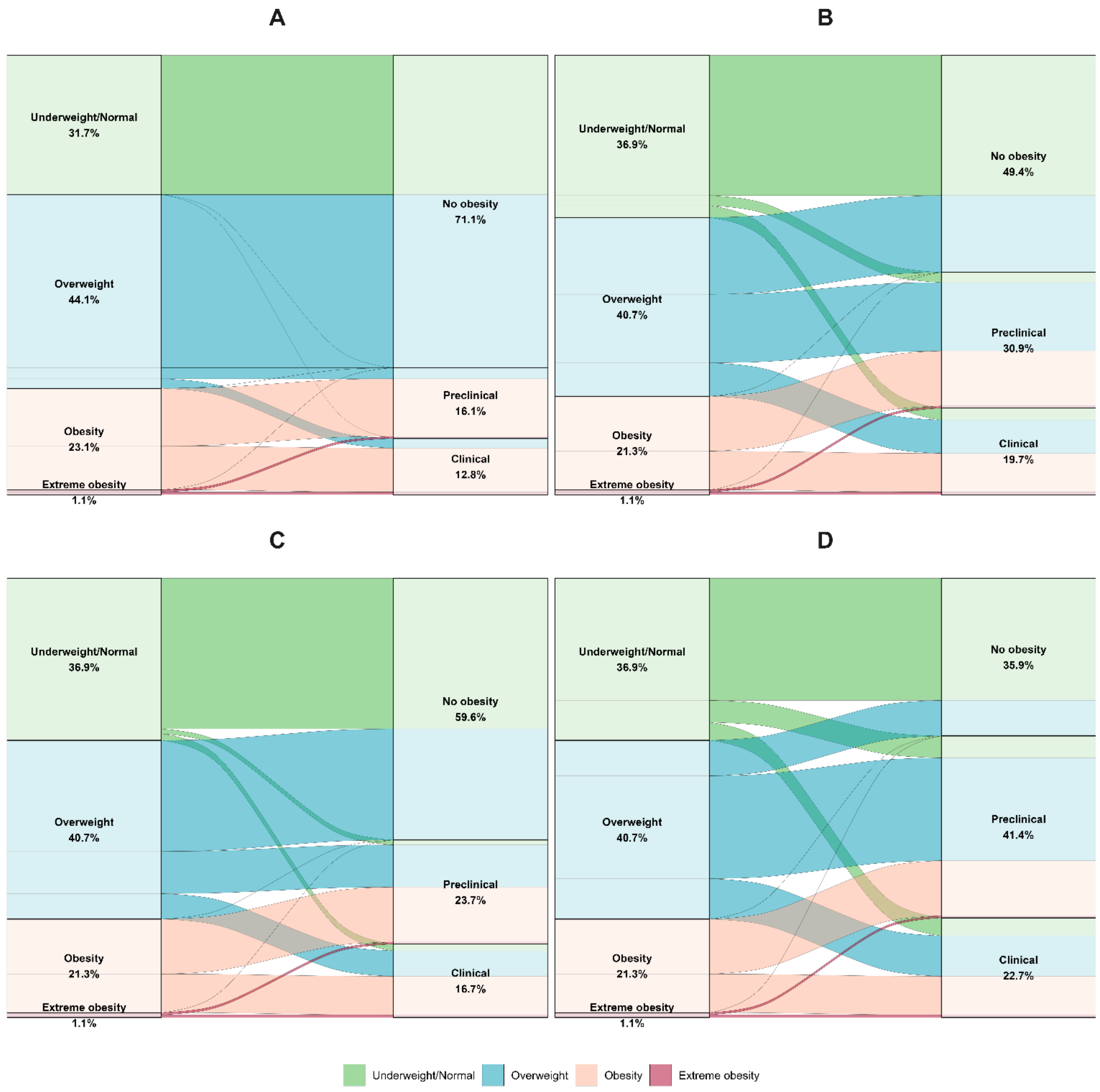

Supplementary Figure S2. Age-adjusted prevalence and reclassification of obesity status among women using BMI-based and using different obesity confirmation approach: the Lancet Diabetes & Endocrinology Commission on Clinical Obesity (1A), Peruvian national guidelines (1B), LASO waist circumference cutoffs (1C), and IDF waist circumference cutoffs (1D).

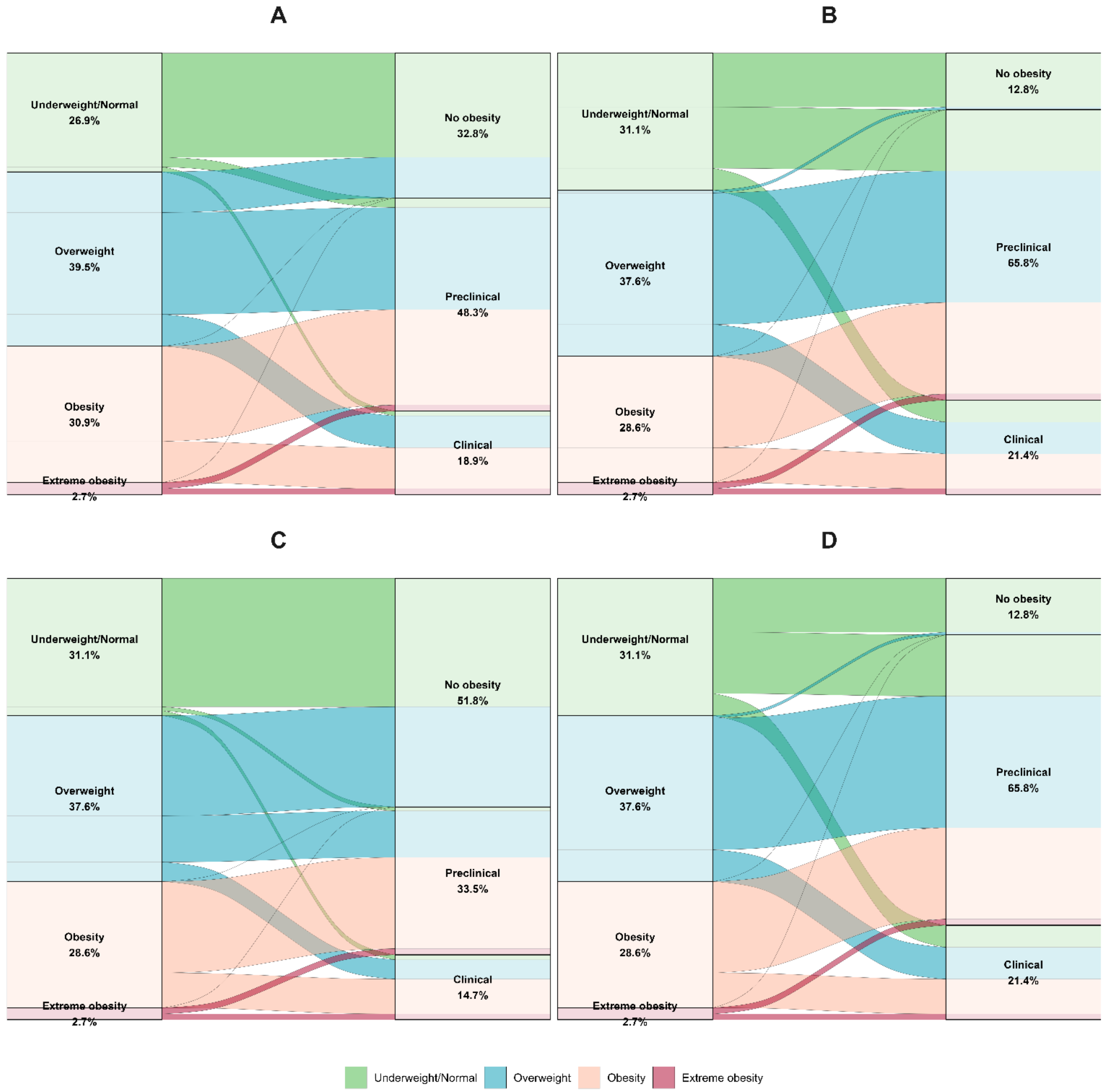

Supplementary Table S1. Geographic distribution of age-adjusted obesity prevalence among Peruvian men and women

Men – Obesity definition based on The Lancet Diabetes & Endocrinology Commission on Clinical Obesity (Obesity confirmation approach 1)

Department	Sex	No obesity	Preclinical obesity	Clinical obesity
Amazonas	Men	84.7	9.6	5.7
Ancash	Men	74.3	15.0	10.7
Apurimac	Men	85.6	8.6	5.8
Arequipa	Men	66.2	16.2	17.5
Ayacucho	Men	82.9	10.1	7.0
Cajamarca	Men	86.3	8.0	5.7
Callao	Men	66.0	18.7	15.4
Cusco	Men	81.4	11.5	7.2
Huancavelica	Men	91.5	4.3	4.2
Huanuco	Men	82.7	9.9	7.4
Ica	Men	61.5	23.7	14.8
Junin	Men	80.3	12.1	7.6
La Libertad	Men	68.8	19.8	11.3
Lambayeque	Men	73.0	15.7	11.3
Lima	Men	65.0	17.8	17.1
Loreto	Men	78.6	11.4	9.9
Madre de Dios	Men	65.0	24.5	10.5
Moquegua	Men	60.8	24.9	14.3
Pasco	Men	84.2	10.1	5.7
Piura	Men	72.0	16.7	11.3
Puno	Men	76.9	13.2	9.9
San Martin	Men	77.5	14.5	8.1

Department	Sex	No obesity	Preclinical obesity	Clinical obesity
Tacna	Men	60.6	22.8	16.6
Tumbes	Men	64.9	20.8	14.4
Ucayali	Men	75.0	20.1	4.9

Men – Obesity definition adapted from Technical Guidelines of the National Institute of Health (Peru) (Obesity confirmation approach 2)

Department	Sex	No obesity	Preclinical obesity	Clinical obesity
Amazonas	Men	68.4	21.6	10.0
Ancash	Men	51.1	32.7	16.2
Apurimac	Men	70.5	20.0	9.5
Arequipa	Men	44.1	32.0	23.9
Ayacucho	Men	65.3	23.4	11.3
Cajamarca	Men	67.7	21.0	11.3
Callao	Men	42.3	33.5	24.2
Cusco	Men	63.5	24.6	12.0
Huancavelica	Men	77.4	13.4	9.2
Huanuco	Men	66.2	22.6	11.2
Ica	Men	42.1	36.7	21.2
Junin	Men	60.1	26.4	13.5
La Libertad	Men	47.9	35.9	16.2
Lambayeque	Men	45.2	35.6	19.2
Lima	Men	42.5	32.4	25.1
Loreto	Men	59.7	23.6	16.7
Madre de Dios	Men	45.7	39.2	15.1
Moquegua	Men	40.4	40.3	19.3
Pasco	Men	67.0	21.4	11.6
Piura	Men	46.9	33.1	19.9
Puno	Men	54.3	29.6	16.1
San Martin	Men	57.6	27.7	14.7
Tacna	Men	37.3	40.1	22.6
Tumbes	Men	44.5	34.0	21.5

Department	Sex	No obesity	Preclinical obesity	Clinical obesity
Ucayali	Men	54.8	36.9	8.2

Men – Obesity definition adapted from The Latin American Consortium of Studies in Obesity (LASO) (Obesity confirmation approach 3)

Department	Sex	No obesity	Preclinical obesity	Clinical obesity
Amazonas	Men	76.7	15.3	8.0
Ancash	Men	62.4	23.5	14.1
Apurimac	Men	78.6	13.8	7.6
Arequipa	Men	53.8	25.2	21.0
Ayacucho	Men	74.5	15.8	9.7
Cajamarca	Men	76.8	13.8	9.4
Callao	Men	52.6	26.8	20.6
Cusco	Men	71.9	18.2	9.8
Huancavelica	Men	84.7	8.1	7.2
Huanuco	Men	73.5	16.5	10.0
Ica	Men	52.1	29.8	18.1
Junin	Men	70.2	18.7	11.2
La Libertad	Men	58.2	27.6	14.2
Lambayeque	Men	59.0	25.4	15.6
Lima	Men	52.5	25.6	21.9
Loreto	Men	69.2	16.8	14.0
Madre de Dios	Men	55.8	31.4	12.8
Moquegua	Men	48.9	33.9	17.2
Pasco	Men	75.7	15.7	8.6
Piura	Men	59.7	24.5	15.8
Puno	Men	63.6	22.7	13.6
San Martin	Men	67.4	21.2	11.5
Tacna	Men	47.8	32.3	20.0
Tumbes	Men	53.6	28.0	18.4

Department	Sex	No obesity	Preclinical obesity	Clinical obesity
Ucayali	Men	65.1	28.7	6.2

Men – Obesity definition adapted from The International Diabetes Federation (IDF) (Obesity confirmation approach 4)

Department	Sex	No obesity	Preclinical obesity	Clinical obesity
Amazonas	Men	54.3	32.7	13.0
Ancash	Men	37.5	43.8	18.6
Apurimac	Men	56.1	32.1	11.8
Arequipa	Men	30.5	42.6	26.9
Ayacucho	Men	51.9	33.5	14.6
Cajamarca	Men	49.8	35.1	15.1
Callao	Men	29.0	43.1	27.9
Cusco	Men	48.5	36.3	15.2
Huancavelica	Men	63.5	24.2	12.3
Huanuco	Men	52.5	33.5	14.0
Ica	Men	31.6	45.1	23.3
Junin	Men	46.6	37.2	16.1
La Libertad	Men	35.6	45.8	18.6
Lambayeque	Men	31.1	46.5	22.4
Lima	Men	29.6	41.8	28.6
Loreto	Men	45.9	33.8	20.3
Madre de Dios	Men	32.7	50.0	17.2
Moquegua	Men	27.3	50.4	22.3
Pasco	Men	52.4	33.0	14.6
Piura	Men	32.7	44.7	22.6
Puno	Men	40.1	40.4	19.4
San Martin	Men	42.5	39.4	18.1
Tacna	Men	24.1	50.6	25.3
Tumbes	Men	30.0	44.8	25.2

Department	Sex	No obesity	Preclinical obesity	Clinical obesity
Ucayali	Men	40.2	49.9	10.0

Women – Obesity definition based on The Lancet Diabetes & Endocrinology Commission on Clinical Obesity (Obesity confirmation approach 1)

Department	Sex	No obesity	Preclinical obesity	Clinical obesity
Amazonas	Women	42.6	43.8	13.6
Ancash	Women	32.7	51.4	15.9
Apurimac	Women	44.6	44.3	11.2
Arequipa	Women	30.2	52.4	17.4
Ayacucho	Women	41.3	46.8	12.0
Cajamarca	Women	41.2	43.7	15.0
Callao	Women	26.2	50.8	22.9
Cusco	Women	39.6	43.6	16.8
Huancavelica	Women	46.2	40.5	13.3
Huanuco	Women	39.1	48.3	12.7
Ica	Women	29.5	53.4	17.1
Junin	Women	36.0	50.5	13.5
La Libertad	Women	32.8	50.2	17.0
Lambayeque	Women	38.8	44.7	16.6
Lima	Women	29.6	48.2	22.3
Loreto	Women	34.6	44.9	20.5
Madre de Dios	Women	25.4	58.7	15.9
Moquegua	Women	24.0	56.0	20.1
Pasco	Women	35.1	52.4	12.6
Piura	Women	31.3	49.1	19.7
Puno	Women	36.1	49.4	14.5
San Martin	Women	35.6	47.8	16.6
Tacna	Women	29.5	52.8	17.7
Tumbes	Women	25.6	51.8	22.6

Department	Sex	No obesity	Preclinical obesity	Clinical obesity
Ucayali	Women	39.9	47.2	12.9

Women – Obesity definition adapted from Technical Guidelines of the National Institute of Health (Peru) (Obesity confirmation approach 2)

Department	Sex	No obesity	Preclinical obesity	Clinical obesity
Amazonas	Women	16.1	67.0	16.9
Ancash	Women	12.8	68.5	18.7
Apurimac	Women	19.1	66.5	14.4
Arequipa	Women	12.3	68.9	18.8
Ayacucho	Women	16.4	68.4	15.2
Cajamarca	Women	15.0	65.7	19.3
Callao	Women	9.2	65.5	25.4
Cusco	Women	19.7	61.6	18.7
Huancavelica	Women	21.8	60.9	17.2
Huanuco	Women	16.8	68.6	14.7
Ica	Women	10.2	70.3	19.5
Junin	Women	15.4	69.7	14.9
La Libertad	Women	11.6	68.4	20.0
Lambayeque	Women	14.4	65.2	20.4
Lima	Women	11.6	63.6	24.8
Loreto	Women	13.0	62.4	24.6
Madre de Dios	Women	8.5	73.1	18.4
Moquegua	Women	7.2	71.5	21.3
Pasco	Women	12.0	73.4	14.6
Piura	Women	9.8	67.3	22.9
Puno	Women	19.4	63.4	17.2
San Martin	Women	12.8	67.2	19.9
Tacna	Women	9.4	71.0	19.6
Tumbes	Women	7.6	67.6	24.8

Department	Sex	No obesity	Preclinical obesity	Clinical obesity
Ucayali	Women	14.3	70.5	15.3

Women – Obesity definition adapted from The Latin American Consortium of Studies in Obesity (LASO) (Obesity confirmation approach 3)

Department	Sex	No obesity	Preclinical obesity	Clinical obesity
Amazonas	Women	63.3	27.1	9.6
Ancash	Women	52.9	34.4	12.8
Apurimac	Women	64.0	28.0	8.0
Arequipa	Women	47.6	37.7	14.6
Ayacucho	Women	60.6	30.6	8.8
Cajamarca	Women	62.5	27.5	10.0
Callao	Women	45.5	36.0	18.5
Cusco	Women	59.2	28.0	12.7
Huancavelica	Women	67.9	23.1	9.0
Huanuco	Women	58.7	31.6	9.8
Ica	Women	46.2	39.1	14.6
Junin	Women	57.9	31.7	10.5
La Libertad	Women	51.7	35.0	13.3
Lambayeque	Women	55.7	30.5	13.8
Lima	Women	48.6	33.8	17.6
Loreto	Women	54.7	30.0	15.3
Madre de Dios	Women	41.9	44.9	13.2
Moquegua	Women	40.1	42.6	17.2
Pasco	Women	55.6	34.2	10.2
Piura	Women	50.2	34.0	15.8
Puno	Women	52.1	36.0	11.9
San Martin	Women	55.7	32.1	12.3
Tacna	Women	45.1	39.7	15.2
Tumbes	Women	44.3	37.9	17.9

Department	Sex	No obesity	Preclinical obesity	Clinical obesity
Ucayali	Women	56.8	33.6	9.7

Women – Obesity definition adapted from The International Diabetes Federation (IDF) (Obesity confirmation approach 4)

Department	Sex	No obesity	Preclinical obesity	Clinical obesity
Amazonas	Women	16.1	67.0	16.9
Ancash	Women	12.8	68.5	18.7
Apurimac	Women	19.1	66.5	14.4
Arequipa	Women	12.3	68.9	18.8
Ayacucho	Women	16.4	68.4	15.2
Cajamarca	Women	15.0	65.7	19.3
Callao	Women	9.2	65.5	25.4
Cusco	Women	19.7	61.6	18.7
Huancavelica	Women	21.8	60.9	17.2
Huanuco	Women	16.8	68.6	14.7
Ica	Women	10.2	70.3	19.5
Junin	Women	15.4	69.7	14.9
La Libertad	Women	11.6	68.4	20.0
Lambayeque	Women	14.4	65.2	20.4
Lima	Women	11.6	63.6	24.8
Loreto	Women	13.0	62.4	24.6
Madre de Dios	Women	8.5	73.1	18.4
Moquegua	Women	7.2	71.5	21.3
Pasco	Women	12.0	73.4	14.6
Piura	Women	9.8	67.3	22.9
Puno	Women	19.4	63.4	17.2
San Martin	Women	12.8	67.2	19.9
Tacna	Women	9.4	71.0	19.6
Tumbes	Women	7.6	67.6	24.8

Department	Sex	No obesity	Preclinical obesity	Clinical obesity
Ucayali	Women	14.3	70.5	15.3

Supplementary Table S2. Age-specific percentiles of waist circumference and waist-to-height ratio by sex and ethnicity.

Supplementary Table 1. Age-specific percentiles of Waist circumference for men by ethnicity.

Age	3rd	50th	97th	Ethnicity
20	68.96	81.02	102.42	Quechua-Aymara
21	69.51	81.80	103.20	Quechua-Aymara
22	70.05	82.59	103.98	Quechua-Aymara
23	70.59	83.37	104.76	Quechua-Aymara
24	71.12	84.16	105.54	Quechua-Aymara
25	71.66	84.94	106.31	Quechua-Aymara
26	72.19	85.71	107.07	Quechua-Aymara
27	72.70	86.46	107.80	Quechua-Aymara
28	73.21	87.20	108.50	Quechua-Aymara
29	73.70	87.90	109.17	Quechua-Aymara
30	74.16	88.56	109.79	Quechua-Aymara
31	74.58	89.18	110.35	Quechua-Aymara
32	74.97	89.74	110.86	Quechua-Aymara
33	75.31	90.24	111.31	Quechua-Aymara
34	75.61	90.69	111.70	Quechua-Aymara
35	75.87	91.10	112.06	Quechua-Aymara
36	76.10	91.45	112.36	Quechua-Aymara
37	76.28	91.75	112.63	Quechua-Aymara
38	76.42	92.00	112.85	Quechua-Aymara
39	76.52	92.21	113.03	Quechua-Aymara
40	76.57	92.36	113.17	Quechua-Aymara

Age	3rd	50th	97th	Ethnicity
41	76.59	92.47	113.29	Quechua-Aymara
42	76.58	92.56	113.39	Quechua-Aymara
43	76.55	92.64	113.48	Quechua-Aymara
44	76.51	92.70	113.59	Quechua-Aymara
45	76.46	92.76	113.70	Quechua-Aymara
46	76.39	92.81	113.82	Quechua-Aymara
47	76.32	92.85	113.94	Quechua-Aymara
48	76.23	92.89	114.07	Quechua-Aymara
49	76.13	92.92	114.20	Quechua-Aymara
50	76.03	92.94	114.33	Quechua-Aymara
51	75.92	92.97	114.48	Quechua-Aymara
52	75.81	92.99	114.63	Quechua-Aymara
53	75.71	93.01	114.80	Quechua-Aymara
54	75.60	93.04	114.98	Quechua-Aymara
55	75.50	93.07	115.17	Quechua-Aymara
56	75.41	93.11	115.37	Quechua-Aymara
57	75.31	93.14	115.56	Quechua-Aymara
58	75.21	93.15	115.75	Quechua-Aymara
59	75.11	93.15	115.91	Quechua-Aymara
60	74.99	93.13	116.04	Quechua-Aymara
61	74.86	93.08	116.13	Quechua-Aymara
62	74.72	93.01	116.19	Quechua-Aymara
63	74.57	92.92	116.20	Quechua-Aymara
64	74.43	92.81	116.16	Quechua-Aymara
65	74.29	92.69	116.10	Quechua-Aymara

Age	3rd	50th	97th	Ethnicity
66	74.16	92.56	116.00	Quechua-Aymara
67	74.04	92.43	115.89	Quechua-Aymara
68	73.94	92.30	115.76	Quechua-Aymara
69	73.86	92.18	115.62	Quechua-Aymara
70	73.79	92.06	115.47	Quechua-Aymara
71	73.72	91.93	115.31	Quechua-Aymara
72	73.67	91.80	115.13	Quechua-Aymara
73	73.62	91.67	114.94	Quechua-Aymara
74	73.56	91.53	114.73	Quechua-Aymara
75	73.50	91.37	114.50	Quechua-Aymara
76	73.43	91.21	114.26	Quechua-Aymara
77	73.36	91.03	114.00	Quechua-Aymara
78	73.28	90.84	113.72	Quechua-Aymara
79	73.18	90.65	113.43	Quechua-Aymara
80	73.08	90.45	113.14	Quechua-Aymara
81	72.97	90.24	112.83	Quechua-Aymara
82	72.85	90.02	112.51	Quechua-Aymara
83	72.72	89.79	112.18	Quechua-Aymara
84	72.58	89.55	111.84	Quechua-Aymara
85	72.42	89.31	111.50	Quechua-Aymara
86	72.27	89.07	111.16	Quechua-Aymara
87	72.10	88.82	110.81	Quechua-Aymara
88	71.93	88.57	110.47	Quechua-Aymara
89	71.75	88.33	110.14	Quechua-Aymara
90	71.57	88.08	109.80	Quechua-Aymara

Age	3rd	50th	97th	Ethnicity
91	71.39	87.83	109.47	Quechua-Aymara
92	71.20	87.58	109.14	Quechua-Aymara
93	71.01	87.33	108.81	Quechua-Aymara
94	70.82	87.08	108.48	Quechua-Aymara
95	70.62	86.83	108.16	Quechua-Aymara
96	70.42	86.58	107.83	Quechua-Aymara
97	70.23	86.33	107.51	Quechua-Aymara
20	69.04	84.38	108.92	Afro-Peruvian
21	69.62	85.08	109.68	Afro-Peruvian
22	70.19	85.77	110.42	Afro-Peruvian
23	70.75	86.44	111.14	Afro-Peruvian
24	71.29	87.09	111.83	Afro-Peruvian
25	71.80	87.70	112.48	Afro-Peruvian
26	72.28	88.29	113.09	Afro-Peruvian
27	72.74	88.84	113.65	Afro-Peruvian
28	73.16	89.35	114.16	Afro-Peruvian
29	73.56	89.82	114.64	Afro-Peruvian
30	73.92	90.26	115.07	Afro-Peruvian
31	74.27	90.68	115.47	Afro-Peruvian
32	74.59	91.07	115.84	Afro-Peruvian
33	74.89	91.44	116.18	Afro-Peruvian
34	75.18	91.79	116.51	Afro-Peruvian
35	75.46	92.13	116.82	Afro-Peruvian
36	75.73	92.45	117.12	Afro-Peruvian
37	75.98	92.77	117.40	Afro-Peruvian

Age	3rd	50th	97th	Ethnicity
38	76.22	93.06	117.67	Afro-Peruvian
39	76.45	93.34	117.92	Afro-Peruvian
40	76.66	93.61	118.15	Afro-Peruvian
41	76.85	93.86	118.36	Afro-Peruvian
42	77.03	94.08	118.55	Afro-Peruvian
43	77.19	94.29	118.72	Afro-Peruvian
44	77.34	94.48	118.87	Afro-Peruvian
45	77.46	94.64	118.98	Afro-Peruvian
46	77.56	94.78	119.07	Afro-Peruvian
47	77.64	94.89	119.14	Afro-Peruvian
48	77.70	94.99	119.17	Afro-Peruvian
49	77.74	95.06	119.18	Afro-Peruvian
50	77.76	95.11	119.17	Afro-Peruvian
51	77.77	95.14	119.14	Afro-Peruvian
52	77.77	95.16	119.10	Afro-Peruvian
53	77.76	95.17	119.04	Afro-Peruvian
54	77.74	95.17	118.97	Afro-Peruvian
55	77.71	95.16	118.89	Afro-Peruvian
56	77.67	95.14	118.81	Afro-Peruvian
57	77.63	95.11	118.71	Afro-Peruvian
58	77.57	95.07	118.60	Afro-Peruvian
59	77.51	95.02	118.48	Afro-Peruvian
60	77.43	94.95	118.33	Afro-Peruvian
61	77.33	94.87	118.17	Afro-Peruvian
62	77.22	94.77	117.99	Afro-Peruvian

Age	3rd	50th	97th	Ethnicity
63	77.10	94.65	117.78	Afro-Peruvian
64	76.96	94.51	117.56	Afro-Peruvian
65	76.81	94.36	117.32	Afro-Peruvian
66	76.65	94.19	117.06	Afro-Peruvian
67	76.49	94.02	116.80	Afro-Peruvian
68	76.31	93.85	116.54	Afro-Peruvian
69	76.14	93.67	116.27	Afro-Peruvian
70	75.98	93.50	116.01	Afro-Peruvian
71	75.81	93.34	115.76	Afro-Peruvian
72	75.66	93.18	115.52	Afro-Peruvian
73	75.51	93.04	115.30	Afro-Peruvian
74	75.36	92.90	115.09	Afro-Peruvian
75	75.23	92.77	114.89	Afro-Peruvian
76	75.10	92.66	114.70	Afro-Peruvian
77	74.98	92.55	114.53	Afro-Peruvian
78	74.87	92.45	114.36	Afro-Peruvian
79	74.75	92.35	114.21	Afro-Peruvian
80	74.65	92.26	114.06	Afro-Peruvian
81	74.54	92.17	113.91	Afro-Peruvian
82	74.43	92.08	113.75	Afro-Peruvian
83	74.31	91.98	113.60	Afro-Peruvian
84	74.19	91.87	113.43	Afro-Peruvian
85	74.06	91.76	113.25	Afro-Peruvian
86	73.92	91.63	113.06	Afro-Peruvian
87	73.77	91.49	112.86	Afro-Peruvian

Age	3rd	50th	97th	Ethnicity
88	73.62	91.34	112.64	Afro-Peruvian
89	73.45	91.18	112.41	Afro-Peruvian
90	73.27	91.01	112.17	Afro-Peruvian
91	73.09	90.84	111.92	Afro-Peruvian
92	72.91	90.65	111.66	Afro-Peruvian
93	72.72	90.46	111.40	Afro-Peruvian
94	72.53	90.27	111.13	Afro-Peruvian
95	72.33	90.08	110.86	Afro-Peruvian
96	72.14	89.88	110.59	Afro-Peruvian
97	71.94	89.69	110.32	Afro-Peruvian
20	67.89	82.78	107.07	Others
21	68.71	83.77	108.09	Others
22	69.53	84.75	109.10	Others
23	70.33	85.72	110.09	Others
24	71.12	86.67	111.06	Others
25	71.88	87.59	111.98	Others
26	72.61	88.47	112.85	Others
27	73.30	89.29	113.64	Others
28	73.92	90.04	114.34	Others
29	74.49	90.72	114.96	Others
30	75.01	91.34	115.50	Others
31	75.48	91.89	115.96	Others
32	75.91	92.40	116.36	Others
33	76.29	92.84	116.69	Others
34	76.63	93.25	116.97	Others

Age	3rd	50th	97th	Ethnicity
35	76.94	93.61	117.21	Others
36	77.22	93.94	117.40	Others
37	77.47	94.24	117.56	Others
38	77.69	94.50	117.69	Others
39	77.88	94.73	117.79	Others
40	78.05	94.94	117.86	Others
41	78.21	95.13	117.92	Others
42	78.34	95.30	117.96	Others
43	78.45	95.45	117.98	Others
44	78.55	95.59	118.01	Others
45	78.64	95.72	118.03	Others
46	78.72	95.85	118.06	Others
47	78.80	95.98	118.09	Others
48	78.87	96.11	118.13	Others
49	78.92	96.22	118.16	Others
50	78.95	96.31	118.17	Others
51	78.96	96.38	118.17	Others
52	78.96	96.44	118.16	Others
53	78.94	96.49	118.15	Others
54	78.91	96.53	118.14	Others
55	78.88	96.57	118.13	Others
56	78.84	96.61	118.12	Others
57	78.79	96.65	118.11	Others
58	78.74	96.68	118.10	Others
59	78.69	96.71	118.10	Others

Age	3rd	50th	97th	Ethnicity
60	78.62	96.74	118.08	Others
61	78.55	96.75	118.06	Others
62	78.46	96.75	118.02	Others
63	78.36	96.73	117.96	Others
64	78.24	96.70	117.88	Others
65	78.11	96.65	117.79	Others
66	77.96	96.58	117.67	Others
67	77.80	96.50	117.53	Others
68	77.62	96.40	117.38	Others
69	77.44	96.28	117.21	Others
70	77.24	96.16	117.02	Others
71	77.03	96.02	116.81	Others
72	76.82	95.87	116.60	Others
73	76.59	95.71	116.37	Others
74	76.36	95.55	116.14	Others
75	76.13	95.39	115.91	Others
76	75.90	95.23	115.68	Others
77	75.67	95.07	115.46	Others
78	75.44	94.91	115.24	Others
79	75.20	94.75	115.02	Others
80	74.96	94.59	114.80	Others
81	74.72	94.43	114.57	Others
82	74.47	94.26	114.35	Others
83	74.21	94.09	114.11	Others
84	73.95	93.91	113.87	Others

Age	3rd	50th	97th	Ethnicity
85	73.67	93.72	113.62	Others
86	73.39	93.52	113.36	Others
87	73.10	93.31	113.09	Others
88	72.80	93.10	112.81	Others
89	72.50	92.87	112.53	Others
90	72.19	92.65	112.23	Others
91	71.87	92.41	111.93	Others
92	71.55	92.18	111.63	Others
93	71.22	91.94	111.32	Others
94	70.90	91.70	111.01	Others
95	70.57	91.46	110.71	Others
96	70.24	91.22	110.40	Others
97	69.91	90.98	110.09	Others

Supplementary Table 2. Age-specific percentiles of Waist circumference for women by ethnicity.

Age	3rd	50th	97th	Ethnicity
20	67.33	82.43	103.29	Quechua-Aymara
21	68.20	83.51	104.45	Quechua-Aymara
22	69.04	84.56	105.57	Quechua-Aymara
23	69.84	85.57	106.65	Quechua-Aymara
24	70.60	86.52	107.66	Quechua-Aymara
25	71.31	87.42	108.59	Quechua-Aymara
26	71.97	88.25	109.45	Quechua-Aymara
27	72.57	89.02	110.22	Quechua-Aymara
28	73.12	89.71	110.89	Quechua-Aymara
29	73.61	90.34	111.49	Quechua-Aymara
30	74.04	90.90	112.01	Quechua-Aymara
31	74.43	91.42	112.48	Quechua-Aymara
32	74.77	91.90	112.92	Quechua-Aymara
33	75.07	92.33	113.32	Quechua-Aymara
34	75.32	92.73	113.70	Quechua-Aymara
35	75.53	93.10	114.07	Quechua-Aymara
36	75.68	93.43	114.41	Quechua-Aymara
37	75.79	93.74	114.75	Quechua-Aymara
38	75.84	94.00	115.07	Quechua-Aymara
39	75.85	94.24	115.37	Quechua-Aymara
40	75.80	94.44	115.66	Quechua-Aymara
41	75.71	94.60	115.94	Quechua-Aymara

Age	3rd	50th	97th	Ethnicity
42	75.56	94.74	116.21	Quechua-Aymara
43	75.36	94.84	116.47	Quechua-Aymara
44	75.11	94.92	116.74	Quechua-Aymara
45	74.81	94.97	117.00	Quechua-Aymara
46	74.47	95.00	117.27	Quechua-Aymara
47	74.08	95.00	117.54	Quechua-Aymara
48	73.66	94.98	117.80	Quechua-Aymara
49	73.21	94.94	118.06	Quechua-Aymara
50	72.73	94.88	118.31	Quechua-Aymara
51	72.23	94.79	118.54	Quechua-Aymara
52	71.72	94.69	118.74	Quechua-Aymara
53	71.21	94.56	118.91	Quechua-Aymara
54	70.70	94.41	119.04	Quechua-Aymara
55	70.20	94.23	119.11	Quechua-Aymara
56	69.72	94.03	119.13	Quechua-Aymara
57	69.27	93.81	119.10	Quechua-Aymara
58	68.83	93.56	119.02	Quechua-Aymara
59	68.41	93.29	118.88	Quechua-Aymara
60	68.00	92.99	118.70	Quechua-Aymara
61	67.60	92.67	118.48	Quechua-Aymara
62	67.21	92.33	118.21	Quechua-Aymara
63	66.83	91.96	117.92	Quechua-Aymara
64	66.45	91.57	117.60	Quechua-Aymara
65	66.08	91.17	117.27	Quechua-Aymara
66	65.72	90.77	116.93	Quechua-Aymara

Age	3rd	50th	97th	Ethnicity
67	65.39	90.38	116.61	Quechua-Aymara
68	65.09	90.01	116.31	Quechua-Aymara
69	64.81	89.64	116.02	Quechua-Aymara
70	64.56	89.29	115.75	Quechua-Aymara
71	64.32	88.96	115.49	Quechua-Aymara
72	64.11	88.63	115.23	Quechua-Aymara
73	63.91	88.31	114.97	Quechua-Aymara
74	63.74	87.99	114.71	Quechua-Aymara
75	63.58	87.68	114.42	Quechua-Aymara
76	63.44	87.36	114.11	Quechua-Aymara
77	63.32	87.04	113.77	Quechua-Aymara
78	63.21	86.71	113.39	Quechua-Aymara
79	63.12	86.38	112.99	Quechua-Aymara
80	63.04	86.04	112.54	Quechua-Aymara
81	62.98	85.70	112.07	Quechua-Aymara
82	62.92	85.35	111.56	Quechua-Aymara
83	62.89	85.00	111.02	Quechua-Aymara
84	62.86	84.64	110.45	Quechua-Aymara
85	62.83	84.27	109.84	Quechua-Aymara
86	62.81	83.90	109.20	Quechua-Aymara
87	62.78	83.51	108.53	Quechua-Aymara
88	62.75	83.11	107.83	Quechua-Aymara
89	62.72	82.70	107.10	Quechua-Aymara
90	62.68	82.29	106.35	Quechua-Aymara
91	62.64	81.86	105.58	Quechua-Aymara

Age	3rd	50th	97th	Ethnicity
92	62.58	81.43	104.79	Quechua-Aymara
93	62.52	81.00	103.99	Quechua-Aymara
94	62.46	80.56	103.18	Quechua-Aymara
95	62.39	80.12	102.37	Quechua-Aymara
96	62.31	79.68	101.56	Quechua-Aymara
97	62.22	79.24	100.75	Quechua-Aymara
20	67.81	85.51	108.56	Afro-Peruvian
21	68.43	86.19	109.19	Afro-Peruvian
22	69.04	86.85	109.81	Afro-Peruvian
23	69.65	87.50	110.40	Afro-Peruvian
24	70.26	88.14	110.97	Afro-Peruvian
25	70.86	88.76	111.51	Afro-Peruvian
26	71.45	89.36	112.02	Afro-Peruvian
27	72.03	89.95	112.49	Afro-Peruvian
28	72.61	90.51	112.93	Afro-Peruvian
29	73.17	91.06	113.34	Afro-Peruvian
30	73.72	91.58	113.72	Afro-Peruvian
31	74.25	92.07	114.06	Afro-Peruvian
32	74.76	92.54	114.38	Afro-Peruvian
33	75.25	92.98	114.66	Afro-Peruvian
34	75.71	93.39	114.92	Afro-Peruvian
35	76.13	93.77	115.15	Afro-Peruvian
36	76.51	94.12	115.37	Afro-Peruvian
37	76.86	94.44	115.57	Afro-Peruvian
38	77.16	94.73	115.77	Afro-Peruvian

Age	3rd	50th	97th	Ethnicity
39	77.42	94.99	115.97	Afro-Peruvian
40	77.63	95.23	116.18	Afro-Peruvian
41	77.79	95.44	116.40	Afro-Peruvian
42	77.90	95.63	116.62	Afro-Peruvian
43	77.97	95.79	116.85	Afro-Peruvian
44	78.01	95.93	117.07	Afro-Peruvian
45	78.00	96.05	117.30	Afro-Peruvian
46	77.96	96.15	117.52	Afro-Peruvian
47	77.90	96.23	117.73	Afro-Peruvian
48	77.82	96.29	117.94	Afro-Peruvian
49	77.72	96.34	118.13	Afro-Peruvian
50	77.60	96.38	118.32	Afro-Peruvian
51	77.46	96.40	118.49	Afro-Peruvian
52	77.31	96.41	118.66	Afro-Peruvian
53	77.15	96.41	118.81	Afro-Peruvian
54	76.98	96.41	118.96	Afro-Peruvian
55	76.79	96.38	119.10	Afro-Peruvian
56	76.59	96.35	119.23	Afro-Peruvian
57	76.37	96.31	119.35	Afro-Peruvian
58	76.13	96.25	119.46	Afro-Peruvian
59	75.88	96.17	119.55	Afro-Peruvian
60	75.61	96.08	119.63	Afro-Peruvian
61	75.33	95.98	119.69	Afro-Peruvian
62	75.04	95.86	119.72	Afro-Peruvian
63	74.74	95.72	119.73	Afro-Peruvian

Age	3rd	50th	97th	Ethnicity
64	74.43	95.57	119.70	Afro-Peruvian
65	74.13	95.40	119.64	Afro-Peruvian
66	73.82	95.22	119.55	Afro-Peruvian
67	73.52	95.02	119.41	Afro-Peruvian
68	73.22	94.81	119.24	Afro-Peruvian
69	72.93	94.59	119.02	Afro-Peruvian
70	72.65	94.35	118.77	Afro-Peruvian
71	72.37	94.09	118.48	Afro-Peruvian
72	72.10	93.83	118.15	Afro-Peruvian
73	71.84	93.56	117.79	Afro-Peruvian
74	71.58	93.27	117.40	Afro-Peruvian
75	71.34	92.98	116.98	Afro-Peruvian
76	71.10	92.69	116.54	Afro-Peruvian
77	70.87	92.39	116.09	Afro-Peruvian
78	70.65	92.08	115.61	Afro-Peruvian
79	70.44	91.78	115.12	Afro-Peruvian
80	70.24	91.47	114.63	Afro-Peruvian
81	70.06	91.17	114.12	Afro-Peruvian
82	69.88	90.87	113.61	Afro-Peruvian
83	69.72	90.58	113.10	Afro-Peruvian
84	69.56	90.29	112.59	Afro-Peruvian
85	69.42	90.01	112.08	Afro-Peruvian
86	69.28	89.73	111.58	Afro-Peruvian
87	69.16	89.46	111.08	Afro-Peruvian
88	69.03	89.20	110.60	Afro-Peruvian

Age	3rd	50th	97th	Ethnicity
89	68.92	88.94	110.12	Afro-Peruvian
90	68.81	88.68	109.64	Afro-Peruvian
91	68.70	88.43	109.18	Afro-Peruvian
92	68.59	88.19	108.72	Afro-Peruvian
93	68.48	87.94	108.26	Afro-Peruvian
94	68.38	87.70	107.81	Afro-Peruvian
95	68.28	87.46	107.37	Afro-Peruvian
96	68.17	87.22	106.92	Afro-Peruvian
97	68.07	86.98	106.49	Afro-Peruvian
20	67.09	82.74	106.52	Others
21	67.90	83.66	107.41	Others
22	68.69	84.57	108.27	Others
23	69.46	85.46	109.11	Others
24	70.21	86.31	109.91	Others
25	70.93	87.13	110.66	Others
26	71.62	87.90	111.37	Others
27	72.26	88.63	112.02	Others
28	72.86	89.31	112.62	Others
29	73.40	89.93	113.15	Others
30	73.90	90.49	113.63	Others
31	74.34	91.00	114.06	Others
32	74.73	91.45	114.43	Others
33	75.06	91.84	114.76	Others
34	75.35	92.20	115.06	Others
35	75.61	92.53	115.34	Others

Age	3rd	50th	97th	Ethnicity
36	75.85	92.85	115.62	Others
37	76.06	93.14	115.90	Others
38	76.25	93.43	116.17	Others
39	76.44	93.71	116.45	Others
40	76.60	93.98	116.73	Others
41	76.76	94.25	117.02	Others
42	76.91	94.50	117.30	Others
43	77.04	94.75	117.58	Others
44	77.17	94.99	117.85	Others
45	77.28	95.22	118.12	Others
46	77.38	95.44	118.37	Others
47	77.46	95.65	118.61	Others
48	77.53	95.83	118.83	Others
49	77.58	96.01	119.03	Others
50	77.62	96.17	119.23	Others
51	77.64	96.31	119.40	Others
52	77.64	96.44	119.56	Others
53	77.63	96.55	119.70	Others
54	77.60	96.63	119.81	Others
55	77.55	96.69	119.89	Others
56	77.47	96.73	119.93	Others
57	77.37	96.73	119.94	Others
58	77.25	96.71	119.92	Others
59	77.10	96.66	119.86	Others
60	76.94	96.59	119.77	Others

Age	3rd	50th	97th	Ethnicity
61	76.77	96.50	119.65	Others
62	76.58	96.39	119.52	Others
63	76.38	96.28	119.37	Others
64	76.17	96.15	119.22	Others
65	75.95	96.02	119.05	Others
66	75.73	95.87	118.87	Others
67	75.49	95.71	118.67	Others
68	75.24	95.54	118.46	Others
69	74.99	95.37	118.24	Others
70	74.73	95.18	118.00	Others
71	74.46	94.98	117.76	Others
72	74.18	94.78	117.50	Others
73	73.90	94.57	117.25	Others
74	73.62	94.36	116.99	Others
75	73.34	94.16	116.73	Others
76	73.06	93.96	116.48	Others
77	72.78	93.76	116.23	Others
78	72.50	93.56	115.99	Others
79	72.22	93.36	115.75	Others
80	71.94	93.17	115.51	Others
81	71.66	92.98	115.28	Others
82	71.37	92.79	115.06	Others
83	71.09	92.61	114.84	Others
84	70.81	92.43	114.62	Others
85	70.53	92.25	114.42	Others

Age	3rd	50th	97th	Ethnicity
86	70.25	92.09	114.23	Others
87	69.98	91.93	114.04	Others
88	69.70	91.77	113.86	Others
89	69.43	91.62	113.69	Others
90	69.17	91.48	113.53	Others
91	68.90	91.34	113.37	Others
92	68.63	91.21	113.22	Others
93	68.37	91.07	113.06	Others
94	68.10	90.94	112.91	Others
95	67.83	90.81	112.76	Others
96	67.56	90.67	112.61	Others
97	67.28	90.54	112.46	Others

Supplementary Table 3. Age-specific percentiles of WHtR (Waist-to-height ratio) for men by ethnicity.

Age	3rd	50th	97th	Ethnicity
20	0.42	0.49	0.61	Quechua-Aymara
21	0.42	0.50	0.62	Quechua-Aymara
22	0.42	0.50	0.62	Quechua-Aymara
23	0.43	0.51	0.63	Quechua-Aymara
24	0.43	0.51	0.63	Quechua-Aymara
25	0.44	0.52	0.64	Quechua-Aymara
26	0.44	0.52	0.64	Quechua-Aymara
27	0.44	0.53	0.65	Quechua-Aymara
28	0.45	0.53	0.65	Quechua-Aymara
29	0.45	0.54	0.66	Quechua-Aymara
30	0.45	0.54	0.66	Quechua-Aymara
31	0.46	0.55	0.67	Quechua-Aymara
32	0.46	0.55	0.67	Quechua-Aymara
33	0.46	0.55	0.67	Quechua-Aymara
34	0.46	0.56	0.68	Quechua-Aymara
35	0.47	0.56	0.68	Quechua-Aymara
36	0.47	0.56	0.68	Quechua-Aymara
37	0.47	0.56	0.68	Quechua-Aymara
38	0.47	0.56	0.68	Quechua-Aymara
39	0.47	0.56	0.68	Quechua-Aymara
40	0.47	0.57	0.68	Quechua-Aymara
41	0.47	0.57	0.69	Quechua-Aymara

Age	3rd	50th	97th	Ethnicity
42	0.47	0.57	0.69	Quechua-Aymara
43	0.47	0.57	0.69	Quechua-Aymara
44	0.47	0.57	0.69	Quechua-Aymara
45	0.47	0.57	0.69	Quechua-Aymara
46	0.47	0.57	0.69	Quechua-Aymara
47	0.47	0.57	0.69	Quechua-Aymara
48	0.47	0.57	0.70	Quechua-Aymara
49	0.47	0.57	0.70	Quechua-Aymara
50	0.47	0.57	0.70	Quechua-Aymara
51	0.47	0.58	0.70	Quechua-Aymara
52	0.47	0.58	0.70	Quechua-Aymara
53	0.47	0.58	0.70	Quechua-Aymara
54	0.47	0.58	0.71	Quechua-Aymara
55	0.47	0.58	0.71	Quechua-Aymara
56	0.47	0.58	0.71	Quechua-Aymara
57	0.47	0.58	0.71	Quechua-Aymara
58	0.47	0.58	0.71	Quechua-Aymara
59	0.47	0.58	0.72	Quechua-Aymara
60	0.47	0.58	0.72	Quechua-Aymara
61	0.47	0.58	0.72	Quechua-Aymara
62	0.47	0.58	0.72	Quechua-Aymara
63	0.47	0.58	0.72	Quechua-Aymara
64	0.47	0.58	0.72	Quechua-Aymara
65	0.47	0.58	0.72	Quechua-Aymara
66	0.47	0.58	0.72	Quechua-Aymara

Age	3rd	50th	97th	Ethnicity
67	0.47	0.58	0.72	Quechua-Aymara
68	0.47	0.58	0.72	Quechua-Aymara
69	0.47	0.58	0.72	Quechua-Aymara
70	0.47	0.58	0.72	Quechua-Aymara
71	0.47	0.58	0.72	Quechua-Aymara
72	0.47	0.58	0.72	Quechua-Aymara
73	0.47	0.59	0.72	Quechua-Aymara
74	0.47	0.59	0.72	Quechua-Aymara
75	0.47	0.59	0.72	Quechua-Aymara
76	0.48	0.59	0.72	Quechua-Aymara
77	0.48	0.59	0.72	Quechua-Aymara
78	0.48	0.59	0.72	Quechua-Aymara
79	0.48	0.59	0.72	Quechua-Aymara
80	0.48	0.59	0.72	Quechua-Aymara
81	0.48	0.58	0.72	Quechua-Aymara
82	0.48	0.58	0.72	Quechua-Aymara
83	0.48	0.58	0.72	Quechua-Aymara
84	0.48	0.58	0.72	Quechua-Aymara
85	0.48	0.58	0.72	Quechua-Aymara
86	0.48	0.58	0.72	Quechua-Aymara
87	0.48	0.58	0.72	Quechua-Aymara
88	0.48	0.58	0.72	Quechua-Aymara
89	0.48	0.58	0.72	Quechua-Aymara
90	0.48	0.58	0.71	Quechua-Aymara
91	0.48	0.58	0.71	Quechua-Aymara

Age	3rd	50th	97th	Ethnicity
92	0.48	0.58	0.71	Quechua-Aymara
93	0.48	0.58	0.71	Quechua-Aymara
94	0.48	0.58	0.71	Quechua-Aymara
95	0.48	0.58	0.71	Quechua-Aymara
96	0.48	0.58	0.71	Quechua-Aymara
97	0.48	0.58	0.71	Quechua-Aymara
20	0.41	0.51	0.65	Afro-Peruvian
21	0.42	0.51	0.66	Afro-Peruvian
22	0.42	0.52	0.66	Afro-Peruvian
23	0.43	0.52	0.67	Afro-Peruvian
24	0.43	0.53	0.67	Afro-Peruvian
25	0.43	0.53	0.67	Afro-Peruvian
26	0.44	0.53	0.68	Afro-Peruvian
27	0.44	0.54	0.68	Afro-Peruvian
28	0.44	0.54	0.68	Afro-Peruvian
29	0.45	0.54	0.69	Afro-Peruvian
30	0.45	0.55	0.69	Afro-Peruvian
31	0.45	0.55	0.69	Afro-Peruvian
32	0.46	0.55	0.69	Afro-Peruvian
33	0.46	0.56	0.70	Afro-Peruvian
34	0.46	0.56	0.70	Afro-Peruvian
35	0.46	0.56	0.70	Afro-Peruvian
36	0.46	0.56	0.70	Afro-Peruvian
37	0.47	0.56	0.70	Afro-Peruvian
38	0.47	0.57	0.70	Afro-Peruvian

Age	3rd	50th	97th	Ethnicity
39	0.47	0.57	0.71	Afro-Peruvian
40	0.47	0.57	0.71	Afro-Peruvian
41	0.47	0.57	0.71	Afro-Peruvian
42	0.47	0.57	0.71	Afro-Peruvian
43	0.48	0.58	0.71	Afro-Peruvian
44	0.48	0.58	0.71	Afro-Peruvian
45	0.48	0.58	0.71	Afro-Peruvian
46	0.48	0.58	0.71	Afro-Peruvian
47	0.48	0.58	0.72	Afro-Peruvian
48	0.48	0.58	0.72	Afro-Peruvian
49	0.48	0.58	0.72	Afro-Peruvian
50	0.48	0.58	0.72	Afro-Peruvian
51	0.48	0.58	0.72	Afro-Peruvian
52	0.48	0.58	0.72	Afro-Peruvian
53	0.48	0.58	0.72	Afro-Peruvian
54	0.48	0.58	0.72	Afro-Peruvian
55	0.48	0.58	0.72	Afro-Peruvian
56	0.48	0.59	0.72	Afro-Peruvian
57	0.48	0.59	0.72	Afro-Peruvian
58	0.48	0.59	0.72	Afro-Peruvian
59	0.48	0.59	0.72	Afro-Peruvian
60	0.48	0.59	0.72	Afro-Peruvian
61	0.48	0.59	0.72	Afro-Peruvian
62	0.48	0.59	0.72	Afro-Peruvian
63	0.48	0.59	0.72	Afro-Peruvian

Age	3rd	50th	97th	Ethnicity
64	0.48	0.59	0.72	Afro-Peruvian
65	0.48	0.59	0.72	Afro-Peruvian
66	0.48	0.59	0.72	Afro-Peruvian
67	0.48	0.59	0.72	Afro-Peruvian
68	0.48	0.59	0.72	Afro-Peruvian
69	0.48	0.59	0.72	Afro-Peruvian
70	0.48	0.59	0.72	Afro-Peruvian
71	0.48	0.59	0.72	Afro-Peruvian
72	0.48	0.59	0.72	Afro-Peruvian
73	0.48	0.59	0.72	Afro-Peruvian
74	0.48	0.59	0.72	Afro-Peruvian
75	0.48	0.59	0.72	Afro-Peruvian
76	0.48	0.59	0.72	Afro-Peruvian
77	0.48	0.59	0.72	Afro-Peruvian
78	0.48	0.59	0.72	Afro-Peruvian
79	0.48	0.59	0.72	Afro-Peruvian
80	0.48	0.59	0.72	Afro-Peruvian
81	0.48	0.59	0.73	Afro-Peruvian
82	0.48	0.59	0.73	Afro-Peruvian
83	0.48	0.59	0.73	Afro-Peruvian
84	0.48	0.59	0.73	Afro-Peruvian
85	0.48	0.59	0.73	Afro-Peruvian
86	0.48	0.59	0.73	Afro-Peruvian
87	0.48	0.59	0.73	Afro-Peruvian
88	0.47	0.59	0.73	Afro-Peruvian

Age	3rd	50th	97th	Ethnicity
89	0.47	0.59	0.73	Afro-Peruvian
90	0.47	0.59	0.73	Afro-Peruvian
91	0.47	0.59	0.73	Afro-Peruvian
92	0.47	0.59	0.73	Afro-Peruvian
93	0.47	0.59	0.73	Afro-Peruvian
94	0.47	0.59	0.73	Afro-Peruvian
95	0.47	0.59	0.73	Afro-Peruvian
96	0.47	0.59	0.73	Afro-Peruvian
97	0.47	0.59	0.73	Afro-Peruvian
20	0.41	0.50	0.63	Others
21	0.41	0.50	0.64	Others
22	0.42	0.51	0.65	Others
23	0.42	0.52	0.65	Others
24	0.43	0.52	0.66	Others
25	0.43	0.53	0.67	Others
26	0.44	0.53	0.67	Others
27	0.44	0.54	0.68	Others
28	0.45	0.54	0.68	Others
29	0.45	0.55	0.69	Others
30	0.45	0.55	0.69	Others
31	0.46	0.56	0.69	Others
32	0.46	0.56	0.69	Others
33	0.46	0.56	0.70	Others
34	0.47	0.56	0.70	Others
35	0.47	0.57	0.70	Others

Age	3rd	50th	97th	Ethnicity
36	0.47	0.57	0.70	Others
37	0.47	0.57	0.70	Others
38	0.47	0.57	0.70	Others
39	0.47	0.57	0.71	Others
40	0.48	0.58	0.71	Others
41	0.48	0.58	0.71	Others
42	0.48	0.58	0.71	Others
43	0.48	0.58	0.71	Others
44	0.48	0.58	0.71	Others
45	0.48	0.58	0.71	Others
46	0.48	0.58	0.71	Others
47	0.48	0.58	0.71	Others
48	0.48	0.58	0.71	Others
49	0.48	0.59	0.71	Others
50	0.48	0.59	0.71	Others
51	0.48	0.59	0.71	Others
52	0.49	0.59	0.71	Others
53	0.49	0.59	0.72	Others
54	0.49	0.59	0.72	Others
55	0.49	0.59	0.72	Others
56	0.49	0.59	0.72	Others
57	0.49	0.59	0.72	Others
58	0.49	0.59	0.72	Others
59	0.49	0.59	0.72	Others
60	0.49	0.59	0.72	Others

Age	3rd	50th	97th	Ethnicity
61	0.49	0.60	0.72	Others
62	0.49	0.60	0.72	Others
63	0.49	0.60	0.72	Others
64	0.49	0.60	0.72	Others
65	0.49	0.60	0.72	Others
66	0.49	0.60	0.72	Others
67	0.49	0.60	0.72	Others
68	0.48	0.60	0.72	Others
69	0.48	0.60	0.72	Others
70	0.48	0.60	0.72	Others
71	0.48	0.60	0.72	Others
72	0.48	0.60	0.72	Others
73	0.48	0.60	0.72	Others
74	0.48	0.60	0.72	Others
75	0.48	0.60	0.72	Others
76	0.48	0.60	0.72	Others
77	0.48	0.60	0.72	Others
78	0.48	0.60	0.72	Others
79	0.48	0.60	0.72	Others
80	0.48	0.60	0.72	Others
81	0.48	0.60	0.72	Others
82	0.48	0.60	0.72	Others
83	0.48	0.60	0.72	Others
84	0.47	0.60	0.72	Others
85	0.47	0.60	0.72	Others

Age	3rd	50th	97th	Ethnicity
86	0.47	0.60	0.72	Others
87	0.47	0.60	0.72	Others
88	0.47	0.60	0.72	Others
89	0.47	0.60	0.72	Others
90	0.47	0.60	0.72	Others
91	0.47	0.60	0.72	Others
92	0.47	0.60	0.72	Others
93	0.47	0.60	0.72	Others
94	0.47	0.60	0.72	Others
95	0.47	0.60	0.72	Others
96	0.46	0.60	0.72	Others
97	0.46	0.60	0.72	Others

Supplementary Table 4. Age-specific percentiles of WHtR (Waist-to-height ratio) for women by ethnicity.

Age	3rd	50th	97th	Ethnicity
20	0.44	0.54	0.68	Quechua-Aymara
21	0.45	0.55	0.69	Quechua-Aymara
22	0.45	0.56	0.69	Quechua-Aymara
23	0.46	0.56	0.70	Quechua-Aymara
24	0.46	0.57	0.71	Quechua-Aymara
25	0.47	0.58	0.71	Quechua-Aymara
26	0.47	0.58	0.72	Quechua-Aymara
27	0.48	0.59	0.73	Quechua-Aymara
28	0.48	0.59	0.73	Quechua-Aymara
29	0.48	0.60	0.73	Quechua-Aymara
30	0.49	0.60	0.74	Quechua-Aymara
31	0.49	0.60	0.74	Quechua-Aymara
32	0.49	0.61	0.74	Quechua-Aymara
33	0.49	0.61	0.75	Quechua-Aymara
34	0.50	0.61	0.75	Quechua-Aymara
35	0.50	0.61	0.75	Quechua-Aymara
36	0.50	0.62	0.76	Quechua-Aymara
37	0.50	0.62	0.76	Quechua-Aymara
38	0.50	0.62	0.76	Quechua-Aymara
39	0.50	0.62	0.76	Quechua-Aymara
40	0.50	0.63	0.77	Quechua-Aymara

Age	3rd	50th	97th	Ethnicity
41	0.50	0.63	0.77	Quechua-Aymara
42	0.50	0.63	0.77	Quechua-Aymara
43	0.50	0.63	0.77	Quechua-Aymara
44	0.50	0.63	0.78	Quechua-Aymara
45	0.50	0.63	0.78	Quechua-Aymara
46	0.50	0.63	0.78	Quechua-Aymara
47	0.50	0.63	0.78	Quechua-Aymara
48	0.49	0.63	0.78	Quechua-Aymara
49	0.49	0.63	0.79	Quechua-Aymara
50	0.49	0.63	0.79	Quechua-Aymara
51	0.49	0.63	0.79	Quechua-Aymara
52	0.48	0.63	0.79	Quechua-Aymara
53	0.48	0.63	0.79	Quechua-Aymara
54	0.48	0.63	0.80	Quechua-Aymara
55	0.48	0.63	0.80	Quechua-Aymara
56	0.47	0.63	0.80	Quechua-Aymara
57	0.47	0.63	0.80	Quechua-Aymara
58	0.47	0.63	0.80	Quechua-Aymara
59	0.47	0.63	0.80	Quechua-Aymara
60	0.46	0.63	0.80	Quechua-Aymara
61	0.46	0.63	0.80	Quechua-Aymara
62	0.46	0.62	0.80	Quechua-Aymara
63	0.46	0.62	0.80	Quechua-Aymara
64	0.45	0.62	0.80	Quechua-Aymara
65	0.45	0.62	0.80	Quechua-Aymara

Age	3rd	50th	97th	Ethnicity
66	0.45	0.62	0.80	Quechua-Aymara
67	0.45	0.62	0.79	Quechua-Aymara
68	0.45	0.61	0.79	Quechua-Aymara
69	0.45	0.61	0.79	Quechua-Aymara
70	0.44	0.61	0.79	Quechua-Aymara
71	0.44	0.61	0.79	Quechua-Aymara
72	0.44	0.61	0.79	Quechua-Aymara
73	0.44	0.61	0.79	Quechua-Aymara
74	0.44	0.61	0.79	Quechua-Aymara
75	0.44	0.61	0.79	Quechua-Aymara
76	0.44	0.60	0.79	Quechua-Aymara
77	0.44	0.60	0.79	Quechua-Aymara
78	0.44	0.60	0.78	Quechua-Aymara
79	0.44	0.60	0.78	Quechua-Aymara
80	0.44	0.60	0.78	Quechua-Aymara
81	0.44	0.60	0.78	Quechua-Aymara
82	0.44	0.60	0.78	Quechua-Aymara
83	0.44	0.60	0.78	Quechua-Aymara
84	0.44	0.60	0.78	Quechua-Aymara
85	0.44	0.60	0.77	Quechua-Aymara
86	0.45	0.59	0.77	Quechua-Aymara
87	0.45	0.59	0.77	Quechua-Aymara
88	0.45	0.59	0.77	Quechua-Aymara
89	0.45	0.59	0.76	Quechua-Aymara
90	0.45	0.59	0.76	Quechua-Aymara

Age	3rd	50th	97th	Ethnicity
91	0.45	0.59	0.76	Quechua-Aymara
92	0.45	0.59	0.75	Quechua-Aymara
93	0.45	0.58	0.75	Quechua-Aymara
94	0.45	0.58	0.75	Quechua-Aymara
95	0.45	0.58	0.74	Quechua-Aymara
96	0.45	0.58	0.74	Quechua-Aymara
97	0.45	0.58	0.74	Quechua-Aymara
20	0.44	0.56	0.70	Afro-Peruvian
21	0.44	0.56	0.71	Afro-Peruvian
22	0.45	0.57	0.71	Afro-Peruvian
23	0.45	0.57	0.72	Afro-Peruvian
24	0.46	0.58	0.72	Afro-Peruvian
25	0.46	0.58	0.73	Afro-Peruvian
26	0.47	0.59	0.73	Afro-Peruvian
27	0.47	0.59	0.73	Afro-Peruvian
28	0.47	0.59	0.74	Afro-Peruvian
29	0.48	0.60	0.74	Afro-Peruvian
30	0.48	0.60	0.74	Afro-Peruvian
31	0.49	0.61	0.75	Afro-Peruvian
32	0.49	0.61	0.75	Afro-Peruvian
33	0.49	0.61	0.75	Afro-Peruvian
34	0.50	0.61	0.75	Afro-Peruvian
35	0.50	0.62	0.76	Afro-Peruvian
36	0.50	0.62	0.76	Afro-Peruvian
37	0.50	0.62	0.76	Afro-Peruvian

Age	3rd	50th	97th	Ethnicity
38	0.51	0.62	0.76	Afro-Peruvian
39	0.51	0.63	0.76	Afro-Peruvian
40	0.51	0.63	0.77	Afro-Peruvian
41	0.51	0.63	0.77	Afro-Peruvian
42	0.51	0.63	0.77	Afro-Peruvian
43	0.51	0.63	0.77	Afro-Peruvian
44	0.51	0.63	0.77	Afro-Peruvian
45	0.51	0.63	0.77	Afro-Peruvian
46	0.51	0.64	0.78	Afro-Peruvian
47	0.51	0.64	0.78	Afro-Peruvian
48	0.51	0.64	0.78	Afro-Peruvian
49	0.51	0.64	0.78	Afro-Peruvian
50	0.51	0.64	0.78	Afro-Peruvian
51	0.51	0.64	0.79	Afro-Peruvian
52	0.51	0.64	0.79	Afro-Peruvian
53	0.51	0.64	0.79	Afro-Peruvian
54	0.51	0.64	0.79	Afro-Peruvian
55	0.51	0.64	0.79	Afro-Peruvian
56	0.51	0.64	0.79	Afro-Peruvian
57	0.51	0.64	0.80	Afro-Peruvian
58	0.51	0.64	0.80	Afro-Peruvian
59	0.51	0.64	0.80	Afro-Peruvian
60	0.51	0.64	0.80	Afro-Peruvian
61	0.50	0.64	0.80	Afro-Peruvian
62	0.50	0.64	0.80	Afro-Peruvian

Age	3rd	50th	97th	Ethnicity
63	0.50	0.64	0.80	Afro-Peruvian
64	0.50	0.64	0.80	Afro-Peruvian
65	0.50	0.64	0.81	Afro-Peruvian
66	0.50	0.64	0.81	Afro-Peruvian
67	0.50	0.64	0.81	Afro-Peruvian
68	0.50	0.64	0.81	Afro-Peruvian
69	0.50	0.64	0.81	Afro-Peruvian
70	0.49	0.64	0.81	Afro-Peruvian
71	0.49	0.64	0.81	Afro-Peruvian
72	0.49	0.64	0.80	Afro-Peruvian
73	0.49	0.64	0.80	Afro-Peruvian
74	0.49	0.64	0.80	Afro-Peruvian
75	0.49	0.64	0.80	Afro-Peruvian
76	0.49	0.64	0.80	Afro-Peruvian
77	0.49	0.64	0.80	Afro-Peruvian
78	0.49	0.64	0.80	Afro-Peruvian
79	0.49	0.63	0.80	Afro-Peruvian
80	0.49	0.63	0.79	Afro-Peruvian
81	0.49	0.63	0.79	Afro-Peruvian
82	0.49	0.63	0.79	Afro-Peruvian
83	0.49	0.63	0.79	Afro-Peruvian
84	0.49	0.63	0.79	Afro-Peruvian
85	0.49	0.63	0.79	Afro-Peruvian
86	0.49	0.63	0.78	Afro-Peruvian
87	0.49	0.63	0.78	Afro-Peruvian

Age	3rd	50th	97th	Ethnicity
88	0.49	0.63	0.78	Afro-Peruvian
89	0.49	0.63	0.78	Afro-Peruvian
90	0.49	0.63	0.78	Afro-Peruvian
91	0.49	0.63	0.78	Afro-Peruvian
92	0.49	0.63	0.78	Afro-Peruvian
93	0.49	0.63	0.78	Afro-Peruvian
94	0.49	0.63	0.77	Afro-Peruvian
95	0.49	0.63	0.77	Afro-Peruvian
96	0.49	0.63	0.77	Afro-Peruvian
97	0.49	0.63	0.77	Afro-Peruvian
20	0.43	0.54	0.69	Others
21	0.44	0.54	0.70	Others
22	0.44	0.55	0.70	Others
23	0.45	0.56	0.71	Others
24	0.45	0.56	0.72	Others
25	0.46	0.57	0.72	Others
26	0.46	0.57	0.73	Others
27	0.47	0.58	0.73	Others
28	0.47	0.58	0.73	Others
29	0.47	0.59	0.74	Others
30	0.48	0.59	0.74	Others
31	0.48	0.59	0.74	Others
32	0.48	0.60	0.75	Others
33	0.49	0.60	0.75	Others
34	0.49	0.60	0.75	Others

Age	3rd	50th	97th	Ethnicity
35	0.49	0.60	0.75	Others
36	0.49	0.61	0.76	Others
37	0.49	0.61	0.76	Others
38	0.50	0.61	0.76	Others
39	0.50	0.61	0.76	Others
40	0.50	0.62	0.77	Others
41	0.50	0.62	0.77	Others
42	0.50	0.62	0.77	Others
43	0.50	0.62	0.77	Others
44	0.50	0.62	0.77	Others
45	0.51	0.63	0.78	Others
46	0.51	0.63	0.78	Others
47	0.51	0.63	0.78	Others
48	0.51	0.63	0.78	Others
49	0.51	0.63	0.78	Others
50	0.51	0.63	0.79	Others
51	0.51	0.63	0.79	Others
52	0.51	0.64	0.79	Others
53	0.51	0.64	0.79	Others
54	0.51	0.64	0.79	Others
55	0.51	0.64	0.79	Others
56	0.51	0.64	0.80	Others
57	0.51	0.64	0.80	Others
58	0.51	0.64	0.80	Others
59	0.51	0.64	0.80	Others

Age	3rd	50th	97th	Ethnicity
60	0.51	0.64	0.80	Others
61	0.51	0.64	0.80	Others
62	0.51	0.64	0.80	Others
63	0.51	0.64	0.80	Others
64	0.51	0.64	0.80	Others
65	0.51	0.64	0.80	Others
66	0.51	0.64	0.80	Others
67	0.51	0.64	0.80	Others
68	0.51	0.64	0.80	Others
69	0.51	0.64	0.80	Others
70	0.50	0.64	0.80	Others
71	0.50	0.64	0.80	Others
72	0.50	0.64	0.80	Others
73	0.50	0.64	0.80	Others
74	0.50	0.64	0.80	Others
75	0.50	0.64	0.80	Others
76	0.50	0.64	0.80	Others
77	0.50	0.64	0.80	Others
78	0.50	0.64	0.80	Others
79	0.50	0.64	0.80	Others
80	0.50	0.64	0.80	Others
81	0.49	0.64	0.80	Others
82	0.49	0.64	0.79	Others
83	0.49	0.64	0.79	Others
84	0.49	0.64	0.79	Others

Age	3rd	50th	97th	Ethnicity
85	0.49	0.64	0.79	Others
86	0.49	0.64	0.79	Others
87	0.49	0.64	0.79	Others
88	0.49	0.64	0.79	Others
89	0.49	0.64	0.79	Others
90	0.49	0.64	0.79	Others
91	0.49	0.64	0.79	Others
92	0.49	0.64	0.79	Others
93	0.48	0.64	0.79	Others
94	0.48	0.64	0.80	Others
95	0.48	0.64	0.80	Others
96	0.48	0.64	0.80	Others
97	0.48	0.64	0.80	Others
